# Comparative efficacy of Chinese patent medicines for non-alcoholic fatty liver disease: A network meta-analysis

**DOI:** 10.3389/fphar.2022.1077180

**Published:** 2023-01-04

**Authors:** Lihui Zhang, Sutong Liu, Yajiao Gu, Shanzheng Li, Minghao Liu, Wenxia Zhao

**Affiliations:** ^1^ The First Clinical Medical College, Henan University of Chinese Medicine, Zhengzhou, China; ^2^ Department of Spleen, Stomach, Hepatobiliary Diseases, The First Affiliated Hospital of Henan University of Chinese Medicine, Zhengzhou, China; ^3^ Zhengzhou Key Laboratory of Traditional Chinese Medicine for Prevention and Treatment of Hepatobiliary Diseases, The First Affiliated Hospital of Henan University of Chinese Medicine, Zhengzhou, China

**Keywords:** non-alcoholic fatty liver disease, Chinese patent medicine (CPM), network meta-analysis, western medicine, efficacy

## Abstract

**Background:** The incidence of Non-alcoholic fatty liver disease (NAFLD) is increasing year by year. Researches showed that Chinese patent medicines (CPMs) had achieved good efficacy in the treatment of Non-alcoholic fatty liver disease. However, the debate on optimum Chinese patent medicine (CPM) persists. Therefore, we conducted a network meta-analysis to objectively compare the efficacy of different Chinese patent medicines in the treatment of Non-alcoholic fatty liver disease.

**Methods:** PubMed, Embase, Cochrane Library, Web of Science, China National Knowledge Infrastructure, Wanfang Database, China Science and Technology Journal Database, and Chinese Biomedical Literature Database were used as databases for RCT researches retrieval. The retrieval time was from establishment of the database to July 2022. After effective data was extracted, Review Manager 5.4 and Cochrane Collaboration System Evaluator’s Manual were used to assess bias risk. STATA 16.0 based on frequency theory was used for the network meta-analysis.

**Results:** Totally 39 studies were included, involving 13 Chinese patent medicines, including 4049 patients, of which 42 patients were lost. In terms of improving clinical efficiency rate, Zhibitai capsule was most likely the best choice of Chinese patent medicine for Non-alcoholic fatty liver disease. Liuwei Wuling tablet had the best effect in reducing serum ALT and AST; Gandan Shukang capsule had the best effect in reducing serum GGT; Qianggan capsule had the best effect in reducing serum TG; Dangfei Liganning capsule had the best effect in reducing serum TC. None of the included studies had serious adverse reactions.

**Conclusion:** For patients with Non-alcoholic fatty liver disease in this NMA, Zhibitai capsule, Liuwei Wuling tablet, Gandan Shukang capsule, Qianggan capsule, Dangfei Liganning capsule might be noteworthy. Due to the uclear risk bias, better designed double-blind, multi center and large sample RCTs are needed which resolve the problems of blinding, selective reporting and allocation concealment.

**Systematic Review Registration:** https://www.crd.york.ac.uk/prospero/, identifier CRD42022341240.

## 1 Introduction

Non-alcoholic fatty liver disease (NAFLD) is complex chronic liver disease linked to being overweight, obesity, insulin resistance. In the past 40 years, NAFLD has become the most common chronic liver disease, with a global incidence rate of 25% and an estimated 3.6 million new cases every year (Shiha et al., 2021). NAFLD is the fastest growing cause of liver related mortality in the world. It has become an important pathogeny of end-stage liver disease, primary liver cancer and liver transplantation. A 51 years cohort study in Sweden reported that even mild fatty liver disease would increase the risk of death by 71%, and the risk was proportional to the severity of fatty liver disease (Sheka et al., 2020).

At present, NAFLD patients are mainly treated with lifestyle interventions, such as diet and exercise (Kamada et al., 2021). However, it is extremely difficult for many NAFLD patients to adhere to these therapies for a long time. In recent years, with the deepening of scholars’ understanding of the pathogenesis of NAFLD, important progress has been made in the research and development of drugs to treat NAFLD (Younossi et al., 2018). Western medicines for NAFLD can be divided into two categories (Fan et al., 2019). The first type is drugs for metabolic syndrome. Such as orlistat, liraglutide, metformin, statins, etc. However, anti-obesity drugs are easy to cause gastrointestinal adverse reactions (Anirban et al., 2018). Metformin, pioglitazone and other drugs can reduce blood sugar and improve insulin resistance, but there was no evidence that hypoglycemic drugs could improve Non-alcoholic steatohepatitis (NASH). Statins can reduce serum low density lipoprotein (LDL) cholesterol levels, but there was no evidence that they could improve NASH and liver fibrosis. The second type is drugs to improve liver injury. Such as vitamin E, silymarin, bicyclol alcohol, polyene phosphatidylcholine, diammonium glycyrrhizinate, reduced glutathione, S-adenosylmethionine, ursodeoxycholic acid, tiopronin, etc. The safety of long-term use of vitamin E is worrying (Traber and Head, 2021). The therapeutic effect of other liver protecting and anti-inflammatory drugs on NASH and liver fibrosis has been unclear, which requires evidence support from large-scale evidence-based medicine. To sum up, although there are many western medicines for NAFLD treatment, their safety and exact effect still need to be confirmed by standardized and large-scale clinical studies. To date, there is no drug approved for NAFLD (Cusi et al., 2022).

Chinese patent medicines (CPMs) such as Qianggan capsule and Danning tablet, which are made of Chinese medicinal materials, also showed good efficacy in the treatment of NAFLD (Liu et al., 2018; Ding et al., 2021). However, there are few studies directly comparing different CPMs in the treatment of NAFLD. It is difficult to evaluate the efficacy of various CPMs in the treatment of NAFLD. A network meta-analysis (NMA) can enhance evidence by combining direct evidence and indirect evidence to compare different interventions. In addition, it also carries out a comprehensive evaluation and ranking of interventions to identify the advantages and disadvantages of various interventions.

In this study, a network meta-analysis technique was used to systematically evaluate the efficacy of a variety of CPMs in the treatment of NAFLD, providing a basis for clinical treatment.

## 2 Methods

This study follows the systematic evaluation of the network meta analysis list and the preferred reporting items of meta analysis (Page et al., 2021). The protocol for the research has been submitted to the International Prospective Register of Systematic Reviews (PROSPERO) (CRD42022341240).

## 3 Eligibility criteria

Inclusion criteria were as follows: 1) Study type: randomized clinical trials (RCTs); 2) Study object: NAFLD patients, refer to the series of guidelines for non-alcoholic fatty liver disease formulated by Fatty Liver and Alcoholic Liver Disease Group of Liver Disease Branch of Chinese Medical Association (National Workshop on Fatty Liver and Alcoholic Liver Disease, Chinese Society of Hepatology, Chinese Medical Association, 2001; National Workshop on Fatty Liver and Alcoholic Liver Disease, Chinese Society of Hepatology, Chinese Medical Association, 2006; National Workshop on Fatty Liver and Alcoholic Liver Disease, Chinese Society of Hepatology, Chinese Medical Association, 2010; National Workshop on Fatty Liver and Alcoholic Liver Disease, Chinese Society of Hepatology, Chinese Medical Association, 2018); 3) Intervention measures: The experiment group was treated with Chinese patent medicine (CPM), while the control group was treated with western medicine, another CPM different from the experiment group, lifestyle modification or placebo. CPMs need to be included in the China Medical Information Platform (https://www.dayi.org.cn/); 4) Outcome indicators: clinical efficiency rate (defined by symptoms, signs, ultrasonic or computed tomography (CT) or other imaging assessment of fatty liver degree, liver function, blood lipids, and comprehensive improvement), alanine aminotransferase (ALT), aspartate aminotransferase (AST), gamma glutamyl transferase (GGT), triglyceride (TG), total cholesterol (TC); 5) Language: only studies in English and Chinese were analysed.

Exclusion criteria were as follows: 1) Patients with other liver diseases such as viral hepatitis; 2) Non-randomized controlled studies, case control studies, experimental studies, case reports, conference summaries, reviews, retrospective studies, meta-analysis; 3) Unable to get full-text; 4) Repeated published researches.

## 4 Search strategy

PubMed, Embase, Cochrane Library, Web of Science, China National Knowledge Infrastructure (CNKI), Wanfang Database, China Science and Technology Journal Database (VIP), and Chinese Biomedical Literature Database (CBM) were used as databases for RCT researches retrieval. The retrieval time was from the establishment of the database to July 2022.

Key words include: Non-alcoholic Fatty Liver Disease, NAFLD, Non-alcoholic Fatty Liver Disease, Fatty Liver, Non-alcoholic, Fatty Livers, Non-alcoholic, Liver, Non-alcoholic Fatty, Livers, Non-alcoholic Fatty, Non-alcoholic Fatty Liver, Non-alcoholic Fatty Livers, Non-alcoholic Steatohepatitis, Non-alcoholic, Steatohepatitis, Non-alcoholic, NASH, non-alcoholic steatohepatitis, metabolic associated fatty liver disease, metabolic associated steatohepatitis, MAFLD, MASH, Proprietary Chinese medicine, Chinese patent medicine, capsule, tablet, pellet, pill, powder.

## 5 Data collection and quality assessment

The characteristics of the included literatures were extracted into Microsoft Excel 2016, including the main author, year of publication, diagnosis, sample size, age, intervention measures, duration, and outcome indicators.

Two authors respectively evaluated the methodological quality of the literatures. In case of disagreement, they would discuss or ask the third senior researcher to make a decision. The quality evaluation included in the studies were conducted according to “Bias Risk Assessment” tool in Handbook 5.1.0 of Cochrane Evaluation Manual: ① Random allocation method; ② Hide allocation scheme; ③ Blind method shall be adopted for subjects and test personnel; ④ The outcome evaluators were blinded; ⑤ Integrity of result data; ⑥ Selective reporting of research results; ⑦ Other sources of bias (such as sample size estimation, baseline comparability, study design, etc.). Make a judgment of “low risk”, “unclear” and “high risk” for the literature.

## 6 Data synthesis and analysis

We used Review Manager 5.4 to draw a Cochrane bias risk map. Stata16.0, JAGS and R (version x64 4.2.1) were used for NMA. We estimated summary odds ratios (ORs) for dichotomous outcomes and mean differences (MD) for continuous outcomes using pairwise and network meta analysis. The significance of an effect was expressed by 95% confidence interval (CI).

All results of the pairwise meta-analysis were described in the tables and forest plots (Cipriani et al., 2018). Network evidence plots were used to show the relationship between interventions. In the network evidence plots, the size of the dot represents the sample size of the treatment method. The larger the dot is, the more the sample size is. The thickness of the line between two points represents the number of studies. The thicker the line is, the more the number of studies is. The Surface Under the Cumulative Ranking (SUCRA) was used to reflect the probability order of different CPMs to be the best treatment option. A higher SUCRA score indicated a more effective or acceptable intervention.

Comparison adjusted funnel plots were used to assess the presence of publication bias. When there was a closed loop, we carried out the inconsistency test. In the inconsistency test, if *p* < 0.05, it was considered that the results of direct comparison and indirect comparison were inconsistent. Global *I* ^*2*^ was used to measure the overall heterogeneity. If *I* ^*2*^ > 75% (Page et al., 2021), it was considered that there was a large heterogeneity. When the degree of heterogeneity was high, we chose random effect network meta-analysis model. Otherwise, the fixed-effects model was selected. Sensitivity analysis was used to evaluate the stability of aggregate effect.

## 7 Results

### 7.1 Search results

**FIGURE 1:**
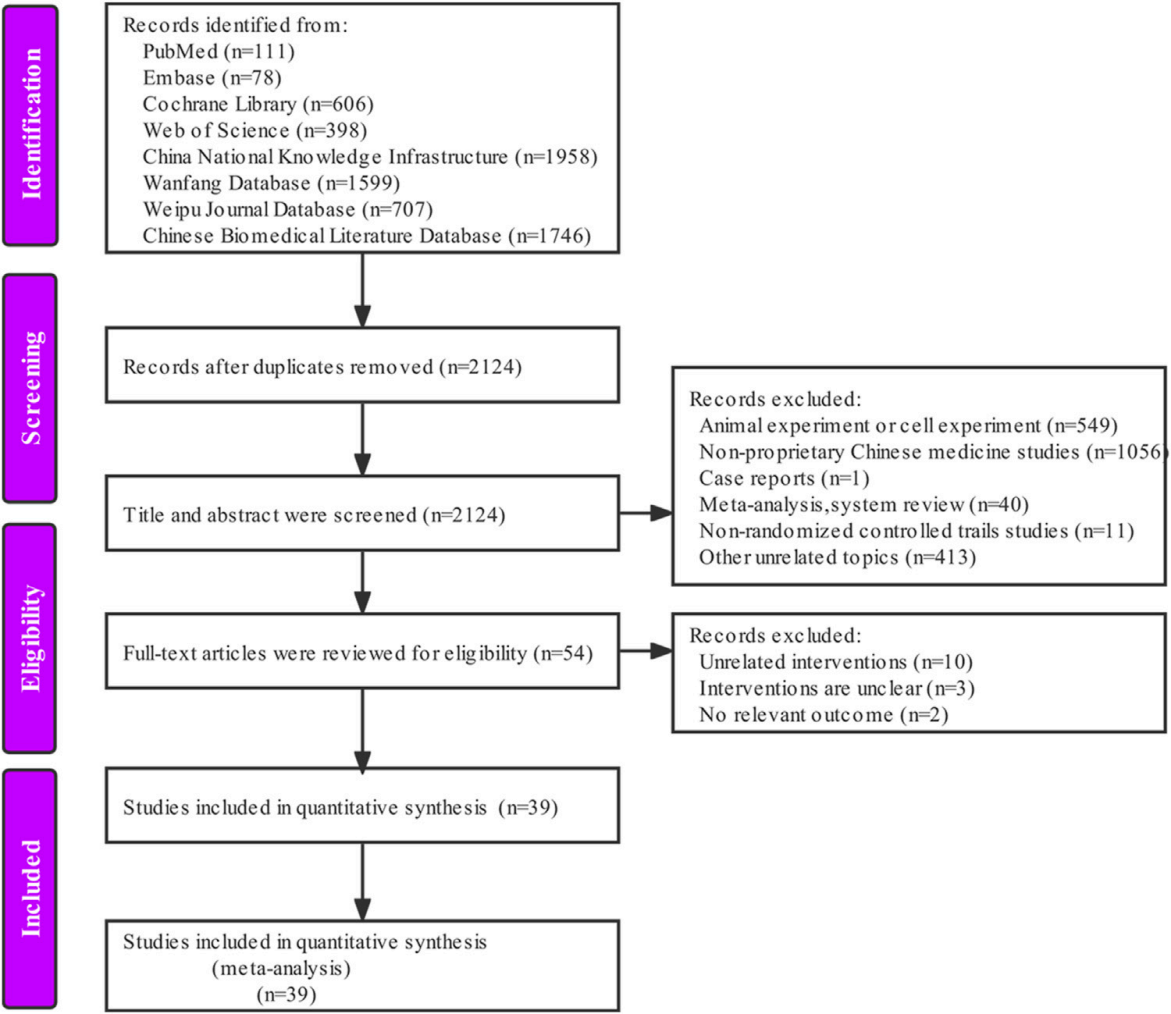
Study selection process.

According to the pre-determined retrieval strategy, 7203 documents were retrieved, and the documents that did not meet the inclusion criteria were excluded by removing duplicates, reading the abstract and full text of the documents. Finally, we included 39 articles (Chen et al., 2006; Huang and Zhang, 2007; Ji et al., 2008; Wu et al., 2008; Yuan, 2008; Li and Jiang, 2009; Lv, 2009; Meng, 2009; Fan et al., 2010; Li et al., 2010; Xu et al., 2010; Deng et al., 2011; Jin, 2011; Liu, 2011; Zhang, 2011; Li et al., 2012; Qi et al., 2012; Lin et al., 2013; Liu and Lv, 2014; Luo and Jiang, 2014; Ma, 2014; Wang, 2014; Yang et al., 2014; Yu et al., 2014; Zhang et al., 2014; Wang et al., 2015; Wei et al., 2015; Yang et al., 2015; He et al., 2016; Ou et al., 2016; Li et al., 2017; Ning et al., 2017; Zhong and Yang, 2017; Wang et al., 2018; Wu and Peng, 2018; Xu and Tao, 2018; Peng, 2019; Zhang, 2019; Nan, 2020). The specific retrieval process is shown in Figure 1.

### 7.2 Characteristics of included studies

The studies included three English studies and 36 Chinese studies, involving 4049 patients, including 42 patients who were not interviewed. The articles were published from 2006 to 2020, and all of them were two arm studies. 13 CPMs were included: Qianggan capsule (6 RCTs), Dangfei Liganning capsule (5 RCTs), Danning tablet (4 RCTs), Huazhi Rougan granule (9 RCTs), Qiaozhi capsule (4 RCTs), Sanqi Zhigan pill (2 RCTs), Liuwei Wuling tablet (3 RCTs), Hedan tablet (2 RCTs), Gandan Shukang capsule (1 RCTs), Xuezhikang capsule (2 RCTs), Yiganling tablet (1 RCTs), Hugan capsule (1 RCTs), Zhibitai capsule (2RCTs). Details characterizations of 13 CPMs are show in Supplementary Table S10. Among them, 27 articles reported clinical efficiency rate (2968 cases), 34 articles reported ALT (3670 cases), 30 articles reported AST (3180 cases), 17 articles reported GGT (1695 cases), 31 articles reported TG (3405 cases), and 29 articles reported TC (3095 cases). The basic characteristics of included studies are shown in Table 1.

**TABLE 1:** Characteristics of included studies.

Study ID	Diagnosis	Diagnostic criteria	Sample size (M/F)	Age (year)	Intervention experimental group	Control group	Duration (weeks)	Outcomes
Chen et al. (2006)	NAFLD	CSH 2001	E: 64 (38/26) C: 58 (39/19)	E: (26 ~ 66) C: (23 ~ 61)	QGJN	WM	12	①②④⑤
Li et al. (2010)	NAFLD	CSH 2006	E:45 (24/21)	E:48.2 ± 12.6 (18 ~ 56) C:44.5 ± 10.8 (26 ~ 61)	QGJN	WM	24	①②③ ④⑤⑥
			C:43 (31/12)					
Ou et al. (2016)	NAFLD	CSH 2010	E:45 (24/21)C:40 (26/14)	E: (34 ~ 65) C: (34 ~ 65)	QGJN	WM	24	②③
He et al. (2016)	NAFLD with Hyperlipidemia	CSH 2010	E:62 (39/23)	E:43 ± 7.4 C:42 ± 6.7	QGJN	WM	24	②③④⑤⑥
			C:50 (30/20)					
Wang et al. (2015)	NAFLD with Hyperlipidemia	CSH 2006	E:64 (44/20) C:64 (42/22)	E:35.8 ± 3.7 (22 ~ 65) C:37.2 ± 3.3 (23 ~ 65)	QGJN	WM	8	①②③④⑤⑥
Liu and Lv, (2014)	NAFLD	CSH 2006	E:70 (44/26)	—	QGJN	WM	24	②③④⑤⑥
			C:62 (42/20)					
Wu and Peng, (2018)	NAFLD	CSH 2010	E:50 (26/24)	E:37.15 ± 1.22 (31 ~ 67) C:36.23 ± 1.56 (30 ~ 65)	DFLGNJN	WM	24	②
			C:50 (25/25)					
Wu et al. (2008)	NAFLD	CSH 2006	E:30 (−/−) C:26 (−/−)	E:38.26 C:38.26	DFLGNJN	WM	12	①②③⑤⑥
Ji et al. (2008)	NAFLD	CSH 2001	E:102 (77/25) C:33 (25/8)	E:48.37 ± 9.6 C:44.43 ± 10.4	DNP	WM	24	①②③④⑤⑥
Lv, (2009)	NAFLD	CSH 2006	E:30 (20/10) C:30 (18/12)	—	DNP	WM	12	①
Wang, (2014)	NAFLD	CSH 2001	E:127 (81/46) C:116 (79/37)	E:46.5 ± 7.5 (27 ~ 63) C:47.9 ± 7.4 (25 ~ 64)	DNP	WM	12	①②③⑤⑥
Ma, (2014)	NAFLD	CSH 2006	E:76 (43/33) C:38 (23/15)	E: (30 ~ 78) C: (40 ~ 80)	DNP	WM	8	①②③⑤⑥
Yang et al. (2015)	NAFLD	CSH 2006	E:90 (60/30) C:90 (58/32)	E:49.5 ± 7.5 (33 ~ 69) C:50.4 ± 7.1 (30 ~ 67)	HZRGKL	WM	12	①②③⑤⑥
Wei et al. (2015)	NAFLD	CSH 2010	E:41 (28/13) C:39 (27/120)	E:43.7 ± 6.4 C:42.9 ± 6.8	HZRGKL	WM	8	②
Li et al. (2017)	NAFLD	CSH 2006	E:57 (30/27) C:58 (32/26)	E:41.3 ± 4.8 C:42.3 ± 5.4	HZRGKL	WM	12	②③④⑤⑥
Wang et al. (2018)	NAFLD	CSH 2006	E:57 (39/18) C:57 (35/22)	E:48.5 ± 8.1 (30 ~ 67) C:49.4 ± 6.1 (32 ~ 70)	HZRGKL	WM	12	①②③⑤⑥
Zhang CM (2019)	NAFLD	CSH 2010	E:30 (19/11) C:30 (18/12)	E:52.0 ± 10.8 (33.5 ~ 70) C:52.3 ± 10.4 (33.9 ~ 70.3)	HZRGKL	WM	/	①
Lin et al. (2013)	NAFLD	CSH 2010	E:60 (42/18) C:60 (48/12)	E:43.3 ± 5.8 (28 ~ 61) C:42.8 ± 6.3 (26 ~ 62)	HZRGKL	WM	12	①②③⑤⑥
Yu et al. (2014)	NAFLD	CSH 2010	E:30 (18/12) C:30 (16/14)	E:44.3 ± 9.3 (24 ~ 63) C:43.7 ± 9.2 (22 ~ 61)	HZRGKL	WM	12	①②③④⑤⑥
Xu and Tao, (2018)	NAFLD	CSH 2006	E:70 (38/32) C:70 (42/28)	E:44.7 ± 7.5 (23 ~ 70) C:43.5 ± 7.1 (28 ~ 68)	HZRGKL	WM	12	①②③⑤⑥
Nan, (2020)	NAFLD	CSH 2018	E:40 (−/−) C:40 (−/−)	E:37.2 ± 9.4 (22 ~ 54) C:37.2 ± 9.4 (22 ~ 54)	HZRGKL	WM	8	①②③④⑤⑥
Meng, (2009)	NAFLD	CSH 2001	E:54 (−/−) C:54 (−/−)	—	QZJN	WM	24	①②③④⑤⑥
Xu et al. (2010)	NAFLD	CSH 2006	E:60 (39/21) C:60 (41/19)	E:42.1 ± 11.7 (20 ~ 61) C:41.7 ± 12.7 (19 ~ 60)	QZJN	WM	12	①②③⑤⑥
Ning et al. (2017)	NAFLD	CSH 2010	E:50 (−/−) C:50 (−/−)	E: (26.6 ~ 57.3) C: (26.6 ~ 57.3)	QZJN	WM	24	②③④⑤⑥
Zhang, (2011)	NAFLD	CSH 2006	E:30 (20/10) C:35 (21/9)	E: (18 ~ 65) C: (18 ~ 67)	SQZGW	WM	12	①⑧
Luo and Jiang, (2014)	NAFLD	CSH 2010	E:59 (37/22) C:57 (34/23)	E:43.8 ± 5.5 (31 ~ 65) C:43.2 ± 5.9 (30 ~ 63)	SQZGW	WM	12	①
Liu, (2011)	NAFLD	CSH 2001	E:26 (−/−)C:20 (−/−)	-	LWWLP	WM	12	①②③④⑤⑥
Zhang et al. (2014)	NAFLD	CSH 2001	E:94 (54/40) C:94 (52/42)	E:58 ± 0.2 (42 ~ 80) C:56 ± 0.4 (40 ~ 78)	LWWLP	WM	12	①②④⑤
Li and Jiang, (2009)	NAFLD	CSH 2001	E:43 (−/−) C:43 (−/−)	E: (20 ~ 68) C: (20 ~ 68)	HDP	WM	12	①②③⑤⑥
Deng et al. (2011)	NAFLD	CSH 2006	E:34 (20/14) C:35 (21/14)	E:48.4 ± 9.9 (21 ~ 65) C:47.9 ± 10.6 (23 ~ 63)	HDP	WM	8	②③④⑤⑥
Yuan, (2008)	NAFLD	CSH 2001	E:34 (25/9) C:34 (22/11)	E:38.22 ± 7.68 C:38.64 ± 8.3	GDSKJN	WM	12	①②③④⑤⑥
Fan et al. (2010)	NAFLD with Hyperlipidemia	CSH 2006	E:37 (−/−) C:40 (−/−)	E:54.62 ± 9.67 C:54.29 ± 10.11	XZKJN	WM	24	②③④⑤⑥
Huang and Zhang, (2007)	NAFLD	CSH 2006	E:32 (18/14) C:10 (6/4)	E:40.21 ± 12.61 (17 ~ 66) C:39.36 ± 14.33 (17 ~ 66)	DFLGNJN	YGLP	12	②③⑤⑥
Qi et al. (2012)	NAFLD	CSH 2006	E:64 (−/−) C:64	E: (16 ~ 64) C: (16 ~ 64)	DFLGNJN	HGJN	12	②③⑤⑥
Yang et al. (2014)	NAFLD	CSH 2010	E:30 (25/5) C:30 (23/7)	E:45 ± 12.3 C:46 ± 15.4	QZJN	XZKJN	24	①②③⑤⑥
Li et al. (2012)	NAFLD	CSH 2006	E:113 (77/36)	E:45.5 ± 11.8 (26 ~ 65) C:46.7 ± 10.8 (26 ~ 65)	DFLGNJN	placebo	12	②③⑤⑥
			C:114 (73/41)					
Peng, (2019)	NAFLD	CSH 2010	E:39 (21/18) C:39 (20/19)	E:39.46 ± 5.47 (26 ~ 64) C:39.89 ± 6.6 (25 ~ 63)	ZBTJN	LM	12	①
Jin, (2011)	NAFLD	CSH 2010	E:35 (−/−) C:32 (−/−)	E:34.7 (18 ~ 65) C:34.7 (18 ~ 65)	LWWLP	LM	12	①②③④⑤⑥
Zhong and Yang, (2017)	NAFLD	CSH 2010	E:40 (30/10) C:40 (32/8)	E:38.5 ± 4.56 (25 ~ 63) C:39.5 ± 4.05 (26 ~ 65)	ZBTJN	LM	12	①②③④⑤⑥

E: Experimental group; C: Control group; CSH, 2001: National Workshop on Fatty Liver and Alcoholic Liver Disease, Chinese Society of Hepatology, Chinese Medical Association, 2001; CSH, 2006: National Workshop on Fatty Liver and Alcoholic Liver Disease, Chinese Society of Hepatology, Chinese Medical Association, 2006; CSH, 2010: National Workshop on Fatty Liver and Alcoholic Liver Disease, Chinese Society of Hepatology, Chinese Medical Association, 2010; CSH, 2018: National Workshop on Fatty Liver and Alcoholic Liver Disease, Chinese Society of Hepatology, Chinese Medical Association, 2018; QGJN: Qianggan capsule; DFLGNJN: Dangfei Liganning capsule; DNP: Danning tablet; HZRGKL: Huazhi Rougan granule; QZJN: Qiaozhi capsule; SQZGW: Sanqi Zhigan pill. LWWLP: Liuwei Wuling tablet; HDP: Hedan tablet; GDSKJN: Gandan Shukang capsule; XZKJN: Xuezhikang capsule; YGLP: Yiganling tablet; HGJN: Hugan capsule; WM: Western medicine; ZBTJN: Zhibitai capsule; LM: lifestyle modification; ①Clinical efficiency rate; ②ALT; ③AST; ④GGT; ⑤TG; ⑥TC.

### 7.3 Risk of bias

**FIGURE 2:**
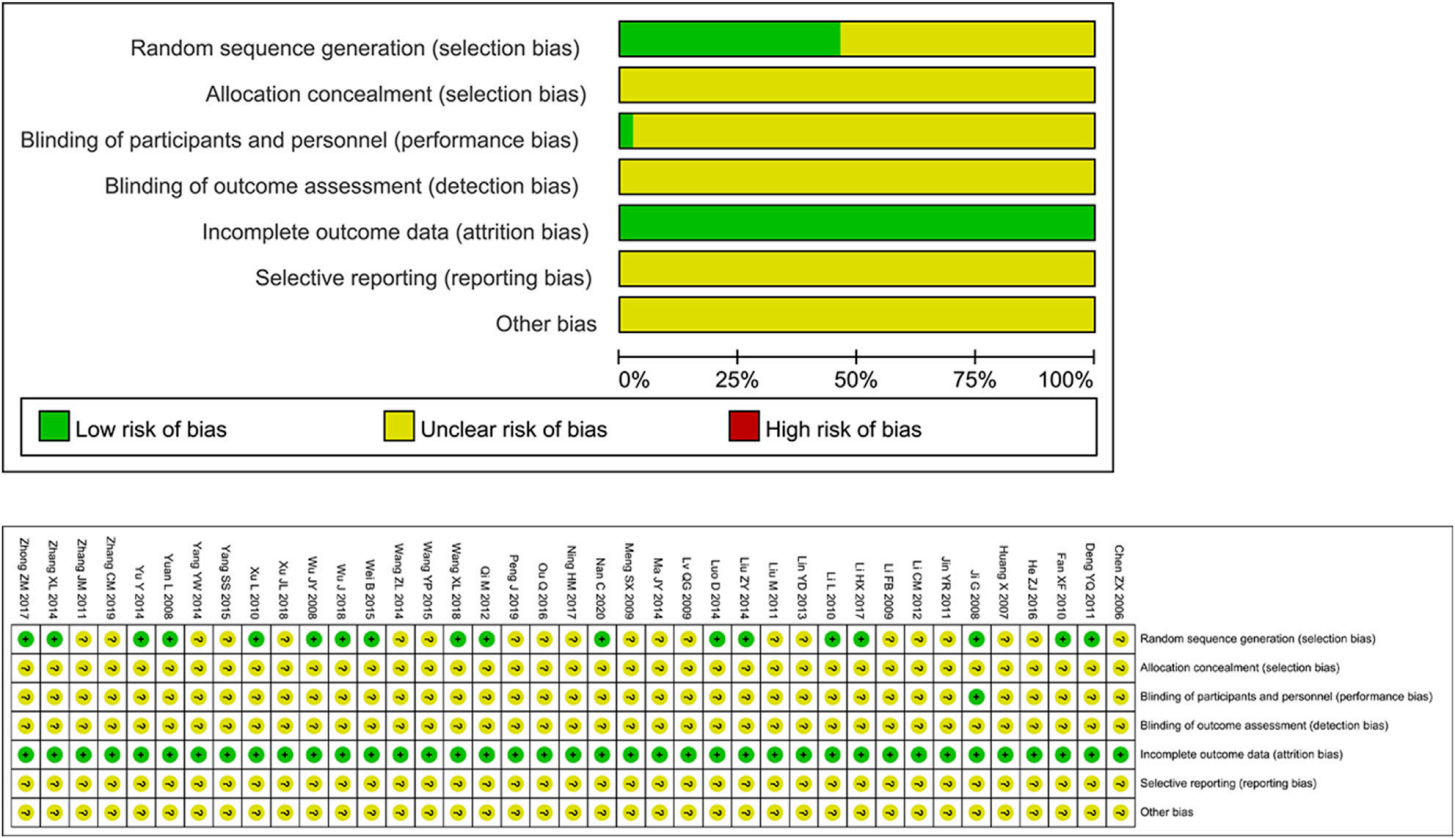
Risk of bias.

The risk assessment of 39 RCTs is shown in Figure 2. Unclear Risk bias were common due to insufficient method reporting. 19 studies used appropriate randomization generation methods, such as the method of generating random numbers or random number tables using computers. All literatures did not indicate the implementation of allocation concealment. One study (Ji et al., 2008) implemented a double-blind method. The data integrity evaluation results were “low risk”, and the selective reporting results and other biases were “unclear".

### 7.4 Network meta-analysis

#### 7.4.1 Primary outcomes

**FIGURE 3:**
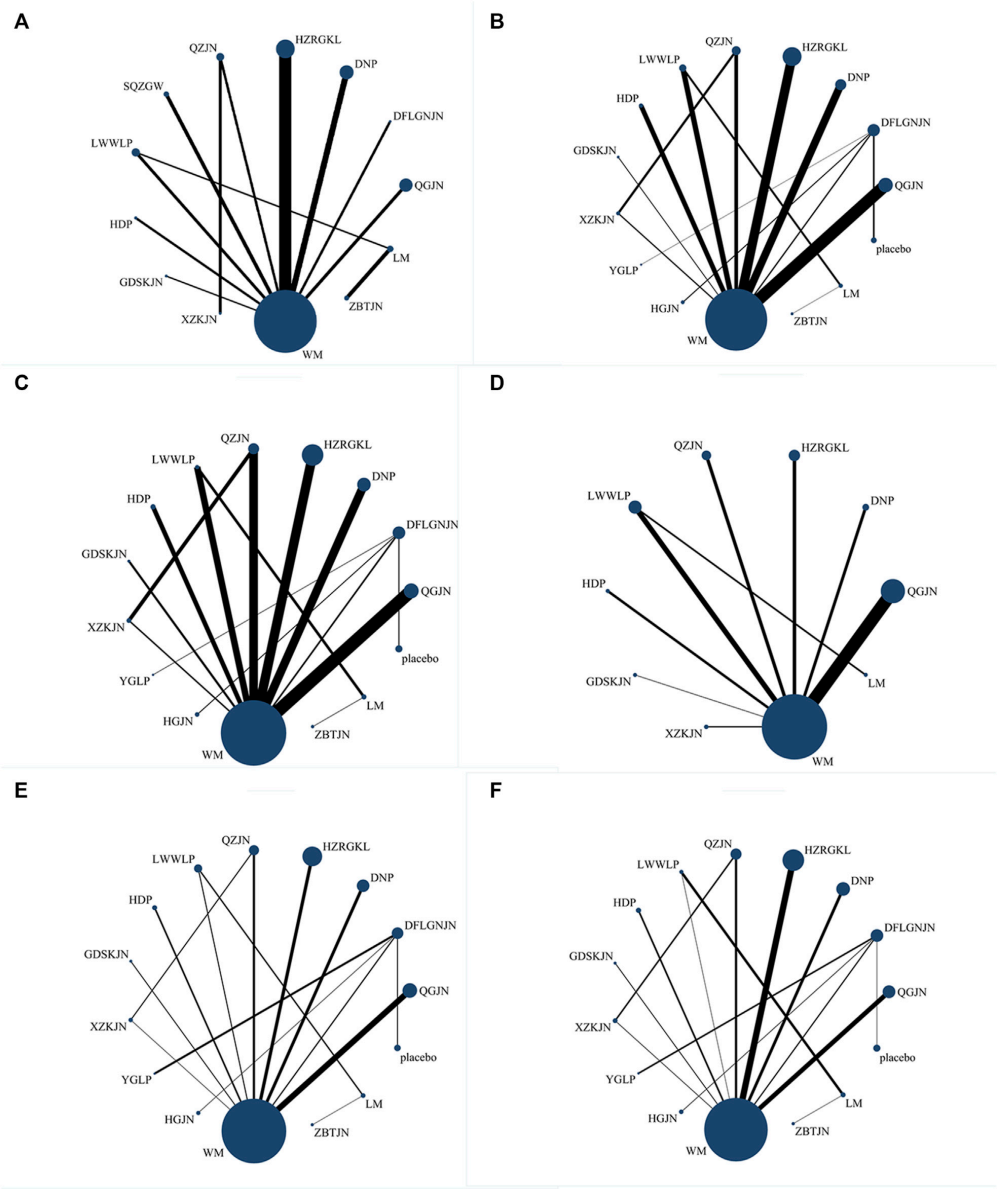
Network evidence plots **(A)** Clinical efficiency rate **(B)** ALT **(C)** AST **(D)** GGT **(E)** TG **(F)** TC; WM, Western medicine; QGJN, Qianggan capsule; DFLGNJN, Dangfei Liganning capsule; DNP, Danning tablet; HZRGKL, Huazhi Rougan granule; QZJN, Qiaozhi capsule; SQZGW, Sanqi Zhigan pill; LWWLP, Liuwei Wuling tablets; HDP, Hedan tablet; GDSKJN, Gandan Shukang capsule; XZKJN, Xuezhikang capsule; YGLP, Yiganling tablet; HGJN, Hugan capsule; ZBTJN, Zhibitai capsule; LM, lifestyle modification.

**FIGURE 4:**
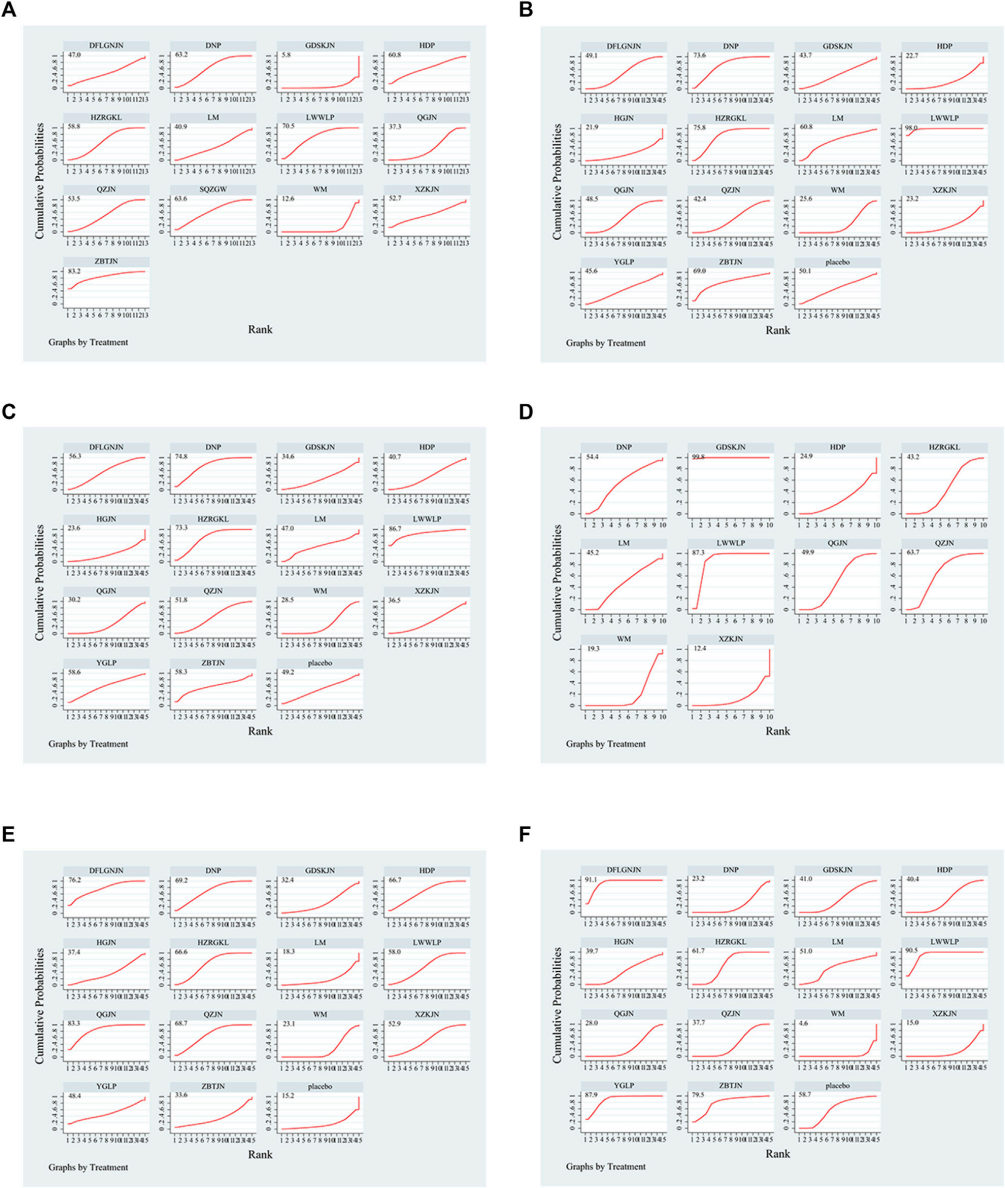
Summary of results from SUCRA **(A)** Clinical efficiency rate **(B)** ALT **(C)** AST **(D)** GGT **(E)** TG **(F)** TC; WM, Western medicine; QGJN, Qianggan capsule; DFLGNJN, Dangfei Liganning capsule; DNP, Danning tablet; HZRGKL, Huazhi Rougan granule; QZJN, Qiaozhi capsule; SQZGW, Sanqi Zhigan pill; LWWLP, Liuwei Wuling tablet; HDP, Hedan tablet; GDSKJN, Gandan Shukang capsule; XZKJN, Xuezhikang capsule; YGLP, Yiganling tablet; HGJN, Hugan capsule; ZBTJN, Zhibitai capsule; LM, lifestyle modification.

27 studies reported clinical efficiency rate, involving 11 CPMs (Qianggan capsule, Dangfei Liganning capsule, Danning tablet, Huazhi Rougan granule, Qiaozhi capsule, Sanqi Zhigan pill, Liuwei Wuling tablet, Hedan tablet, Gandan Shukang capsule, Xuezhikang capsule, Zhibitai capsule). The network relationship between the interventions is shown in Figure 3. In terms of the clinical efficiency rate, according to OR and 95%CI between all the pairwise interventions, Zhibitai capsule (OR: 0.24; 95%CI: 0.07, 0.82) was better than lifestyle modification. Qianggan capsule (OR: 0.47; 95%CI: 0.22, 0.96), Danning tablet (OR: 0.26; 95%CI: 0.12, 0.54), Huazhi Rougan granule (OR: 0.28; 95%CI: 0.16, 0.51), Qiaozhi capsule (OR: 0.32; 95%CI: 0.12, 0.83), Sanqi Zhigan pill (OR: 0.24; 95%CI: 0.08, 0.78), Liuwei Wuling tablet (OR: 0.21; 95%CI: 0.08, 0.57), Zhibitai capsule (OR: 9.38; 95%CI: 1.06, 82.96) were better than western medicine. Danning tablet (OR: 0.14; 95%CI: 0.03, 0.68), Huazhi Rougan granule (OR: 0.15; 95%CI: 0.03, 0.71), Qiaozhi capsule (OR: 0.17; 95%CI: 0.03, 0.95), Sanqi Zhigan pill (OR: 0.13; 95%CI: 0.02, 0.82), Liuwei Wuling tablet (OR: 0.11; 95%CI: 0.02, 0.64), Zhibitai capsule (OR: 17.77; 95%CI: 1.31, 241.28) were better than Gandan Shukang capsule. As shown in Table 2 and Supplementary Figure S1. Moreover, Zhibitai capsule with the highest-ranking probability of SUCRA (83.2%), had the best effectiveness in improving clinical efficiency rate, followed by Liuwei Wuling tablet (70.5%) and Sanqi Zhigan pill (63.6%). More details about the rank probability of SUCRA are shown in Figure 4.

**TABLE 2:** The league table of Clinical efficiency rate and ALT.

Comparisons for clinical efficiency rate (bottom left) and ALT (upper right) of CPMs															
QGJN	−0.33 (−25.17,24.50)	−8.48 (−32.77,15.80)	−8.53 (−30.49,13.43)	1.76 (−21.60,25.12)	—	−25.87 (−51.76,0.03)	8.11 (−17.58,33.81)	1.27 (−27.09,29.64)	7.90 (−17.87,33.67)	0.47 (−30.84,31.77)	9.67 (−21.84,41.18)	5.81 (−15.03,26.65)	−10.10 (−48.07,27.86)	−5.87 (−38.76,27.03)	−1.13 (−33.04,30.77)
1.18 (0.13,10.53)	DFLGNJN	−8.15 (−34.72,18.42)	−8.20 (−32.66,16.26)	2.09 (−23.63.27.82)	—	−25.54 (−53.61,2.54)	8.45 (−19.42.36.31)	1.61 (−28.74,31.95)	8.23 (−19.71.36.18)	0.80 (−26.23.27.83)	10.00 (−17.27,37.27)	6.14 (−17.31.29.59)	−9.77 (−49.28,29.74)	−5.53 (−40.19,29.13)	−0.80 (−28.52.26.92)
1.81 (0.63,5.17)	1.53 (0.17,13.67)	DNP	−0.05 (−23.93,23.83)	10.24 (−14.92.35.41)	—	−17.39 (−44.94,10.17)	16.60 (−10.75,43.94)	9.76 (−20.11.39.62)	16.38 (−11.05,43.82)	8.95 (−23.76,41.66)	18.15 (−14.75,51.05)	14.29 (−8.54,37.12)	−1.62 (−40.77,37.53)	2.62 (−31.62,36.86)	7.35 (−25.93,40.63)
1.64 (0.64,4.19)	1.38 (0.16,11.80)	0.91 (0.35,2.37)	HZRGKL	10.29 (−12.64,33.23)	—	−17.34 (−42.88,8.20)	16.65 (−8.66,41.95)	9.81 (−18.21,37.82)	16.43 (−8.96.41.83)	9.00 (−22.02,40.02)	18.20 (−13.03,49.43)	14.34 (−6.00,34.68)	−1.57 (−39.33,36.19)	2.67 (−29.98.35.31)	7.40 (−24.23.39.03)
1.45 (0.44,4.82)	1.23 (0.13,11.86)	0.80 (0.24,2.70)	0.89 (0.29,2.72)	QZJN	—	**−27.63(−54.38, −0.88)**	6.35 (−20.18,32.88)	−0.49 (−29.61,28.64)	6.14 (−18.45,30.73)	−1.29 (−33.32,30.74)	7.91 (−24.32,40.14)	4.05 (−17.80,25.90)	−11.87 (−50.45,26.72)	−7.63 (−41.23.25.97)	−2.89 (−35.51,29.72)
1.90 (0.48,7.50)	1.61 (0.15,17.10)	1.05 (0.26,4.19)	1.16 (0.32,4.27)	1.31 (0.29,5.90)	SQZGW	—	—	—	—	—	—	—	—	—	—
2.22 (0.64,7.68)	1.87 (0.19,18.52)	1.23 (0.35,4.25)	1.35 (0.42,4.34)	1.53 (0.38,6.11)	1.16 (0.25,5.41)	LWWLP	**33.98(5.17,62.79)**	27.14 (−4.07,58.35)	**33.77(4.88,62.66)**	26.34 (−7.60,60.28)	**35.54(1.41,69.67)**	**31.68(7.11,56.24)**	15.76 (−18.10,49.63)	20.00 (−8.01,48.02)	24.74 (−9.76,59.23)
1.85 (0.24,14.57)	1.57 (0.09,26.32)	1.03 (0.13,8.12)	1.13 (0.15,8.50)	1.28 (0.15,10.99)	0.97 (0.10,9.26)	0.84 (0.10,7.36)	HDP	−6.84 (−37.87,24.19)	−0.21 (−28.90,28.47)	−7.64 (−41.41,26.12)	1.56 (−32.40,35.51)	−2.30 (−26.63,22.02)	−18.22 (−58.26,21.82)	−13.98 (−49.24,21.28)	−9.24 (−43.57,25.08)
0.25 (0.05,1.23)	0.21 (0.02,2.55)	**0.14 (0.03,0.68)**	**0.15 (0.03,0.71)**	**0.17 (0.03,0.95)**	**0.13 (0.02,0.82)**	**0.11 (0.02,0.64)**	0.13 (0.01,1.46)	GDSKJN	6.63 (−24.47,37.73)	−0.80 (−36.65,35.04)	8.40 (−27.62,44.42)	4.54 (−22.59,31.66)	−11.38 (−53.18,30.43)	−7.14 (−44.39,30.11)	−2.40 (−38.77,33.96)
1.45 (0.11,18.97)	1.23 (0.05,30.44)	0.80 (0.06,10.56)	0.89 (0.07,11.17)	1.00 (0.10,9.71)	0.76 (0.05,11.64)	0.65 (0.05,9.38)	0.78 (0.03,17.90)	5.91 (0.34,102.30)	XZKJN	−7.43 (−41.27,26.40)	1.77 (−32.26,35.79)	−2.09 (−26.51,22.33)	−18.00 (−58.11,22.10)	−13.77 (−49.09,21.56)	−9.03 (−43.42,25.36)
—	—	—	—	—	—	—	—	—	—	YGLP	9.20 (−24.10,42.50)	5.34 (−24.89,35.57)	−10.57 (−54.45,33.31)	−6.33 (−45.90,33.23)	−1.60 (−35.27,32.07)
—	—	—	—	—	—	—	—	—	—	—	HGJN	−3.86 (−34.30,26.58)	−19.77 (−63.80,24.25)	−15.53 (−55.26,24.19)	−10.80 (−44.66,23.06)
**0.47 (0.22,0.96)**	0.39 (0.05,3.08)	**0.26 (0.12,0.54)**	**0.28 (0.16,0.51)**	**0.32 (0.12,0.83)**	**0.24 (0.08,0.78)**	**0.21 (0.08,0.57)**	0.25 (0.04,1.73)	1.89 (0.45,7.94)	0.32 (0.03,3.77)	—	—	WM	−15.91 (−53.02,21.19)	−11.67 (−43.56,20.21)	−6.94 (−37.79,23.91)
4.36 (0.44,43.49)	3.68 (0.18,73.94)	2.41 (0.24,24.07)	2.66 (0.28,25.48)	3.01 (0.28,32.48)	2.29 (0.19,27.11)	1.97 (0.28,13.62)	2.35 (0.13,43.22	**17.77 (1.31,241.28)**	3.01 (0.11,80.76)	—	—	**9.38 (1.06,82.96)**	ZBTJN	4.24 (−22.79,31.27)	8.97 (−35.33,53.28)
1.06 (0.15,7.45)	0.89 (0.06,13.87)	0.59 (0.08,4.13)	0.65 (0.10,4.34)	0.73 (0.09,5.64)	0.56 (0.06,4.78)	0.48 (0.11,2.15)	0.57 (0.04,8.04)	4.31 (0.43,43.35)	0.73 (0.03,15.52)	—	—	2.27 (0.37,13.90)	**0.24 (0.07,0.82)**	LM	4.73 (−35.30,44.77)
—										—	—	—	—	—	placebo

QGJN: Qianggan capsule; DFLGNJN: Dangfei Liganning capsule; DNP: Danning tablet; HZRGKL: Huazhi Rougan granule; QZJN: Qiaozhi capsule; SQZGW: Sanqi Zhigan pill. LWWLP: Liuwei Wuling tablet; HDP: Hedan tablet; GDSKJN: Gandan Shukang capsule; XZKJN: Xuezhikang capsule; YGLP: Yiganling tablet; HGJN: Hugan capsule; WM: Western medicine; ZBTJN: Zhibitai capsule; LM: lifestyle modification. Data of comparison for Clinical efficiency rate are OR (95%CI). The 95% confidence interval which does not range across one favors the column-defining treatment and is shown in bold. Data of comparison for ALT, are MD (95%CI). The 95% confidence interval which does not range across 0 favors the column-defining treatment and is shown in bold.

34 studies reported ALT, involving 12 CPMs (Qianggan capsule, Dangfei Liganning capsule, Danning tablet, Huazhi Rougan granule, Qiaozhi capsule, Liuwei Wuling tablet, Hedan tablet, Gandan Shukang capsule, Xuezhikang capsule, Yiganling tablet, Hugan capsule, Zhibitai capsule). The network relationship between the interventions is shown in Figure 3. In terms of ALT improvement, according to MD and 95%CI between all the pairwise interventions, Liuwei Wuling tablet (MD: 31.68; 95%CI: 7.11, 56.24) was superior to western medicine. In addition, Liuwei Wuling tablet was superior to Qiaozhi capsule (MD: −27.63; 95%CI: −54.38, −0.88), Hedan tablet (MD: 33.98; 95%CI: 5.17, 62.79), Xuezhikang capsule (MD: 33.77; 95%CI: 4.88, 62.66), Hugan capsule (MD: 35.54; 95%CI: 1.41, 69.67), as shown in Table 2 and Supplementary Figure S2. Moreover, Liuwei Wuling tablet, with the highest-ranking probability of SUCRA (98.0%), had the best effectiveness in reducing ALT, followed by Huazhi Rougan granule (75.8%) and Danning tablet (73.6%). More details about the rank probability of SUCRA are shown in Figure 4.

#### 7.4.2 Secondary outcomes

30 studies reported AST, involving 12 CPMs (Qianggan capsule, Dangfei Liganning capsule, Danning tablet, Huazhi Rougan granule, Qiaozhi capsule, Liuwei Wuling tablet, Hedan tablet, Gandan Shukang capsule, Xuezhikang capsule, Yiganling tablet, Hugan capsule, Zhibitai capsule). The network relationship between the interventions is shown in Figure 3. According to MD and 95%CI between all the pairwise interventions, in terms of AST improvement, there was no significant difference between 12 CPMs and western medicine. In addition, there was no significant difference between 12 CPMs, as shown in Table 3 and Supplementary Figure S3. However, Liuwei Wuling tablet, with the highest-ranking probability of SUCRA (86.7%), had the best effectiveness in reducing AST, followed by Danning tablet (74.8%). More details about the rank probability of SUCRA are shown in Figure 4.

**TABLE 3:** The league table of AST and GGT.

Comparisons for AST (bottom left) and GGT (upper right) of CPMs														
QGJN	—	2.53 (−16.86,21.91)	−1.67 (−11.80,8.46)	4.29 (−8.08,16.65)	**17.31 (4.45,30.16)**	−7.39 (−25.31,10.54)	**34.72 (21.13,48.31)**	−11.01 (−25.26,3.24)	—	—	−7.09 (−14.18,0.01)	—	−0.42 (−19.29,18.44)	−
−5.76 (−27.20,15.68)	DFLGNJN	—	—	—	—	—	—	—	—	—	—	—	—	—
−10.25 (−28.51,8.02)	−4.49 (−26.86,17.88)	DNP	−4.20 (−23.67,15.28)	1.76 (−18.81,22.34)	14.78 (−6.54,36.10)	−9.91 (−34.36,14.53)	**32.19 (10.74,53.65)**	−13.54 (−35.43,8.35)	—	—	−9.61 (−27.67,8.44)	—	−2.95 (−28.35,22.45)	—
−9.26 (−25.86,7.34)	−3.50 (−24.53,17.53)	0.98 (−16.76,18.73)	HZRGKL	5.96 (−6.33,18.25)	**18.98 (5.55,32.40)**	−5.71 (−23.73,12.30)	**36.39 (22.70,50.09)**	−9.34 (−23.70,5.02)	—	—	−5.41 (−12.70,1.88)	—	1.25 (−18.01,20.51)	—
−4.45 (−22.41,13.51)	1.31 (−20.80,23.42)	5.80 (−13.22,24.82)	4.81 (−12.60,22.23)	QZJN	13.02 (−2.46,28.49)	−11.68 (−30.88,7.53)	**30.43 (15.22,45.64)**	−15.30 (−31.12,0.52)	—	—	**−11.38 (−21.24,−1.51)**	—	−4.71 (−25.44,16.02)	—
−17.86 (−46.14,10.42)	−12.10 (−43.24,19.04)	−7.61 (−36.63,21.40)	−8.60 (−36.60,19.40)	−13.41 (−42.23,15.41)	LWWLP	**−24.69 (−44.69,−4.70)**	**17.41 (1.18,33.65)**	**−28.32 (−45.09,−11.55)**	—	—	**−24.39 (−35.73,−13.06)**	—	**−17.73 (−31.56,−3.90)**	—
−2.10 (−21.24,17.04)	3.66 (−19.43,26.75)	8.15 (−11.99,28.28)	7.16 (−11.47,25.80)	2.35 (−17.50,22.20)	15.76 (−13.81,45.33)	HDP	**42.11 (21.96,62.25)**	−3.62 (−24.23,16.98)	—	—	0.30 (−16.18,16.78)	—	6.96 (−17.33,31.26)	—
−0.26 (−22.22,21.70)	5.50 (−19.97,30.98)	9.99 (−12.85,32.83)	9.00 (−12.52,30.53)	4.19 (−18.39,26.78)	17.60 (−13.87,49.08)	1.84 (−21.70,25.38)	GDSKJN	**−45.73 (−62.68,−28.78)**	—	—	**−41.81 (−53.40,−30.22)**	—	**−35.14 (−56.45,−13.83)**	—
−1.05 (−20.68,18.59)	4.72 (−18.80,28.23)	9.20 (−11.42,29.82)	8.22 (−10.95,27.38)	3.40 (−15.40,22.20)	16.82 (−13.09,46.72)	1.05 (−20.34,22.45)	−0.79 (−24.74,23.17)	XZKJN	—	—	3.92 (−8.45,16.30)	—	10.59 (−11.13,32.31)	—
−6.88 (−32.50,18.74)	−1.12 (−20.95,18.72)	3.37 (−23.03,29.77)	2.38 (−22.89,27.66)	−2.43 (−28.61,23.75)	10.98 (−23.17,45.13)	−4.78 (−31.79,22.23)	−6.62 (−35.70,22.46)	−5.83 (−33.21,21.54)	YGLP	—	—	—	—	—
3.25 (−22.45,28.96)	9.01 (−10.93,28.96)	13.50 (−12.99,39.98)	12.51 (−12.85,37.88)	7.70 (−18.57,33.97)	21.11 (−13.10,55.33)	5.35 (−21.74,32.44)	3.51 (−25.65,32.66)	4.30 (−23.16,31.75)	10.13 (−14.27,34.53)	HGJN	—	—	—	—
0.14 (−15.52,15.81)	5.90 (−14.39,26.20)	10.39 (−6.47,27.25)	9.40 (−5.64,24.44)	4.59 (−11.93,21.11)	18.00 (−9.45,45.45)	2.24 (−15.56,20.04)	0.40 (−20.41,21.21)	1.19 (−17.16,19.54)	7.02 (−17.65,31.69)	−3.11 (−27.87,21.65)	WM	—	—	—
−7.90 (−43.15,27.35)	−2.14 (−39.73,35.45)	2.35 (−33.51,38.20)	1.36 (−33.67,36.39)	−3.45 (−39.15,32.24)	9.96 (−15.54,35.46)	−5.80 (−42.11,30.50)	−7.64 (−45.51,30.23)	−6.86 (−43.44,29.72)	−1.02 (−41.14,39.10)	−11.15 (−51.33,29.02)	−8.04 (−42.64,26.55)	ZBTJN	—	—
−4.18 (−36.56,28.21)	1.58 (−33.33,36.50)	6.07 (−26.97,39.11)	5.08 (−27.07,37.23)	0.27 (−32.60,33.13)	13.68 (−7.63,34.99)	−2.08 (−35.61,31.45)	−3.92 (−39.14,31.30)	−3.13 (−36.96,30.69)	2.70 (−34.93,40.33)	−7.43 (−45.12,30.26)	−4.32 (−35.99,27.35)	3.72 (−16.10,23.55)	LM	—
−4.35 (−30.08,21.39)	1.41 (−18.57,21.40)	5.90 (−20.62,32.42)	4.91 (−20.48,30.31)	0.10 (−26.20,26.40)	13.51 (−20.73,47.75)	−2.25 (−29.37,24.87)	−4.09 (−33.28,25.09)	−3.30 (−30.79,24.18)	2.53 (−21.90,26.96)	−7.60 (−32.12,16.92)	−4.49 (−29.28,20.30)	3.55 (−36.64,43.75)	−0.17 (−37.88,37.54)	placebo

QGJN: Qianggan capsule; DFLGNJN: Dangfei Liganning capsule; DNP: Danning tablet; HZRGKL: Huazhi Rougan granule; QZJN: Qiaozhi capsule; SQZGW: Sanqi Zhigan pill. LWWLP: Liuwei Wuling tablet; HDP: Hedan tablet; GDSKJN: Gandan Shukang capsule; XZKJN: Xuezhikang capsule; YGLP: Yiganling tablet; HGJN: Hugan capsule; WM: Western medicine; ZBTJN: Zhibitai capsule; LM: lifestyle modification. Data of comparison for AST, and GGT, are MD (95%CI).The 95% confidence interval which does not range across 0 favors the column-defining treatment and is shown in bold.

17 studies reported GGT, involving 8 CPMs (Qianggan capsule, Danning tablet, Huazhi Rougan granule, Qiaozhi capsule, Liuwei Wuling tablet, Hedan tablet, Gandan Shukang capsule, Xuezhikang capsule). The network relationship between the interventions is shown in Figure 3. In terms of GGT improvement, according to MD and 95%CI between all the pairwise interventions, Liuwei Wuling tablet (MD: −17.73; 95%CI: −31.56, −3.90) and Gandan Shukang capsule (MD: −35.14; 95%CI: −56.45, −13.83) were superior to lifestyle modification. Qiaozhi capsule (MD: −11.38; 95%CI: −21.24, −1.51), Liuwei Wuling tablet (MD: −24.39; 95%CI: −35.73, −13.06) and Gandan Shukang capsule (MD: −41.81; 95%CI: −53.40, −30.22) were superior to western medicine. In addition, Gandan Shukang capsule was superior to Qianggan capsule (MD: 34.72; 95%CI: 21.13, 48.31), Danning tablet (MD: 32.19; 95%CI: 10.74, 53.65), Huazhi Rougan granule (MD: 36.39; 95%CI: 22.70, 50.09), Qiaozhi capsule (MD: 30.43; 95%CI: 15.22, 45.64), Liuwei Wuling tablet (MD: 17.41; 95%CI: 1.18, 33.65), Hedan tablet (MD: 42.11; 95%CI: 21.96, 62.25), Xuezhikang capsule (MD: −45.73; 95%CI: −62.68, −28.78). Liuwei Wuling tablet was superior to Qianggan capsule (MD: 17.31; 95%CI: 4.45, 30.16), Huazhi Rougan granule (MD: 18.98; 95%CI: 5.55, 32.40), Hedan tablet (MD: −24.69; 95%CI: −44.69, −4.70), Xuezhikang capsule (MD: −28.32; 95%CI: −45.09, −11.55). The detailed results of pairwise comparison are shown in Table 3 and Supplementary Figure S4. Moreover, Gandan Shukang capsule, with the highest-ranking probability of SUCRA (99.8%), had the best effectiveness in reducing GGT, followed by Liuwei Wuling tablet (87.3%) and Qiaozhi capsule (63.7%). More details about the rank probability of SUCRA are shown in Figure 4.

31 studies reported TG, involving 12 CPMs (Qianggan capsule, Dangfei Liganning capsule, Danning tablet, Huazhi Rougan granule, Qiaozhi capsule, Liuwei Wuling tablet, Hedan tablet, Gandan Shukang capsule, Xuezhikang capsule, Yiganling tablet, Hugan capsule, Zhibitai capsule). The network relationship between the interventions is shown in Figure 3. According to MD and 95%CI between all the pairwise interventions, in terms of TG improvement, Qianggan capsule (MD: 0.63; 95%CI: 0.04, 1.22)) was superior to western medicine, as shown in Table 4 and Supplementary Figure S5. Moreover, Qianggan capsule, with the highest-ranking probability of SUCRA (83.3%), had the best effectiveness in reducing TG, followed by Dangfei Liganning capsule (76.2%) and Danning tablet (69.2%). More details about the rank probability of SUCRA are shown in Figure 4.

**TABLE 4:** The league table of TG and TC.

Comparisons for TG (bottom left) and TC (upper right) of CPMs														
QGJN	**−1.59 (−2.31,−0.87)**	0.06 (−0.40,0.52)	**−0.46 (−0.84,−0.08)**	−0.12 (−0.54,0.30)	**−1.55 (−2.07,−1.03)**	−0.16 (−0.64,0.33)	−0.17 (−0.75,0.40)	0.18 (−0.32,0.68)	**−1.50 (−2.58,−0.42)**	−0.19 (−1.02,0.64)	0.31 (−0.01,0.63)	−1.18 (−2.57,0.21)	−0.47 (−1.81,0.86)	−0.51 (−1.34,0.32)
0.03 (−0.94,0.99)	DFLGNJN	**1.65 (0.93,2.38)**	**1.13 (0.45,1.81)**	**1.47 (0.77,2.18)**	0.04 (−0.73,0.81)	**1.44 (0.69,2.18)**	**1.42 (0.62,2.22)**	**1.77 (1.02,2.52)**	0.09 (−0.71,0.89)	**1.40 (0.98,1.82)**	**1.90 (1.25,2.55)**	0.41 (−1.09,1.91)	1.12 (−0.33,2.57)	**1.08 (0.67,1.49)**
0.14 (−0.58,0.85)	0.11 (−0.89,1.10)	DNP	**−0.52 (−0.91,−0.14)**	−0.18 (−0.60,0.25)	**−1.61 (−2.14,−1.08)**	−0.22 (−0.71,0.27)	−0.23 (−0.81,0.35)	0.12 (−0.38,0.62)	**−1.56 (−2.64,−0.48)**	−0.25 (−1.09,0.58)	0.25 (−0.08,0.58)	−1.24 (−2.63,0.15)	−0.53 (−1.87,0.81)	−0.57 (−1.41,0.26)
0.17 (−0.45,0.79)	0.14 (−0.79,1.07)	0.03 (−0.63,0.70)	HZRGKL	0.35 (0.00,0.69)	**−1.09 (−1.55,−0.63)**	0.31 (−0.11,0.73)	0.29 (−0.23,0.81)	**0.64 (0.21,1.08)**	−1.04 (−2.09,0.01)	0.27 (−0.52,1.07)	**0.77 (0.57,0.98)**	−0.72 (−2.09,0.65)	−0.01 (−1.32,1.30)	−0.05 (−0.84,0.74)
0.15 (−0.52,0.81)	0.12 (−0.84,1.08)	0.01 (−0.70,0.73)	−0.02 (−0.64,0.60)	QZJN	**−1.43 (−1.93,−0.94)**	−0.04 (−0.50,0.42)	−0.05 (−0.61,0.50)	0.30 (−0.13,0.72)	**−1.38 (−2.45,−0.32)**	−0.07 (−0.89,0.74)	**0.43 (0.15,0.70)**	−1.06 (−2.44,0.32)	−0.35 (−1.68,0.97)	−0.39 (−1.21,0.42)
0.25 (−0.45,0.95)	0.22 (−0.76,1.20)	0.11 (−0.62,0.85)	0.08 (−0.57,0.73)	0.10 (−0.60.0.80)	LWWLP	**1.40 (0.84,1.95)**	**1.38 (0.75,2.01)**	**1.73 (1.17,2.29)**	0.05 (−1.06,1.16)	**1.36 (0.49,2.23)**	**1.86 (1.45,2.27)**	0.37 (−0.92,1.66)	1.08 (−0.15,2.31)	**1.04 (0.17,1.91)**
0.16 (−0.56,0.88)	0.13 (−0.87,1.14)	0.03 (−0.74,0.79)	−0.01 (−0.69,0.67)	0.01 (−0.71.0.74)	−0.09 (−0.84,0.66)	HDP	−0.02 (−0.62,0.59)	0.34 (−0.19,0.86)	**−1.35 (−2.44,−0.25)**	−0.04 (−0.89,0.82)	**0.46 (0.10,0.83)**	−1.03 (−2.43,0.38)	−0.32 (−1.66,1.03)	−0.36 (−1.20,0.49)
0.56 (−0.30,1.42)	0.53 (−0.57,1.63)	0.42 (−0.47,1.32)	0.39 (−0.43,1.21)	0.41 (−0.45.1.27)	0.31 (−0.57,1.19)	0.40 (−0.51,1.30)	GDSKJN	0.35 (−0.26,0.96)	**−1.33 (−2.47,−0.19)**	−0.02 (−0.93,0.89)	0.48 (0.00,0.96)	−1.01 (−2.44,0.42)	−0.30 (−1.68,1.08)	−0.34 (−1.24,0.56)
0.30 (−0.43,1.03)	0.27 (−0.74.1.27)	0.16 (−0.60,0.93)	0.13 (−0.55,0.81)	0.15 (−0.52,0.82	0.05 (−0.70,0.80)	0.14 (−0.64,0.92)	−0.26 (−1.17,0.65)	XZKJN	**−1.68 (−2.78,−0.58)**	−0.37 (−1.23,0.49)	0.13 (−0.25,0.51)	−1.36 (−2.77,0.04)	−0.65 (−2.00,0.70)	−0.69 (−1.55,0.16)
0.38 (−1.12,1.88)	0.35 (−0.89,1.59)	0.24 (−1.27,1.76)	0.21 (−1.26,1.68)	0.23 (−1.27,1.73)	0.13 (−1.38.1.64)	0.22 (−1.31.1.74)	−0.18 (−1.77.1.41)	0.08 (−1.44,1.60)	YGLP	**1.31 (0.40,2.22)**	**1.81 (0.78,2.84)**	0.32 (−1.38,2.02)	1.03 (−0.63,2.69)	**0.99 (0.09,1.89)**
0.52 (−0.58,1.62)	0.49 (−0.23,1.21)	0.38 (−0.74,1.51)	0.35 (−0.72,1.42)	0.37 (−0.73,1.47)	0.27 (−0.84,1.38)	0.36 (−0.78,1.49)	−0.04 (−1.26.1.18)	0.22 (−0.91,1.36)	0.14 (−1.21,1.49)	HGJN	0.50 (−0.27,1.27)	−0.99 (−2.55,0.57)	−0.28 (−1.79,1.23)	−0.32 (−0.90,0.26)
**0.63(0.04,1.22)**	0.60 (−0.31,1.51)	0.49 (−0.14,1.13)	0.46 (−0.07.0.99)	0.48 (−0.10.1.07)	0.38 (−0.24,0.99)	0.47 (−0.18,1.12)	0.07 (−0.73,0.87)	0.33 (−0.32,0.98)	0.25 (−1.21.1.71)	0.11 (−0.94,1.16)	WM	**−1.49 (−2.84,−0.14)**	−0.78 (−2.08,0.52)	**−0.82 (−1.59,−0.05)**
0.63 (−0.56,1.82)	0.60 (−0.78,1.98)	0.49 (−0.72,1.71)	0.46 (−0.70.1.62)	0.48 (−0.71,1.67)	0.38 (−0.70,1.46)	0.47 (−0.75,1.69)	0.07 (−−1.24,1.38)	0.33 (−0.89.1.56)	0.25 (−1.54,2.04)	0.11 (−1.36,1.58)	0.00 (−1.14,1.14)	ZBTJN	**0.71 (0.32,1.10)**	0.67 (−0.88,2.22)
0.83 (−0.24,1.90)	0.80 (−0.47,2.08)	0.69(−0.40,1.79)	0.66 (−0.38.1.70)	0.68 (−0.39.1.75)	0.58 (−0.37,1.53)	0.67 (−0.44.1.78)	0.27 (−0.93.1.47)	0.53 (−0.58,1.64)	0.45 (−1.26,2.16)	0.31 (−1.07,1.69)	0.20 (−0.82,1.22)	0.20 (−0.51,0.91)	LM	−0.04 (−1.55,1.47)
0.92 (−0.26,2.09	**0.89(0.06,1.72)**	0.78 (−0.41,1.98)	0.75 (−0.40,1.90)	0.77 (−0.40.1.94)	0.67 (−0.52,1.86)	0.76 (−0.45,1.97)	0.36 (−0.93,1.65)	0.62 (−0.59,1.83)	0.54 (−0.87,1.95)	0.40 (−0.58,1.38)	0.29 (−0.84,1.42)	0.29 (−1.24,1.82)	0.09 (−1.35,1.53)	Placebo

QGJN: Qianggan capsule; DFLGNJN: Dangfei Liganning capsule; DNP: Danning tablet; HZRGKL: Huazhi Rougan granule; QZJN: Qiaozhi capsule; SQZGW: Sanqi Zhigan pill. LWWLP: Liuwei Wuling tablet; HDP: Hedan tablet; GDSKJN: Gandan Shukang capsule; XZKJN: Xuezhikang capsule; YGLP: Yiganling tablet; HGJN: Hugan capsule; WM: Western medicine; ZBTJN: Zhibitai capsule; LM: lifestyle modification. Data of comparison for TG, and TC, are MD (95%CI). The 95% confidence interval which does not range across 0 favors the column-defining treatment and is shown in bold.

29 studies reported TC, involving 12 CPMs (Qianggan capsule, Dangfei Liganning capsule, Danning tablet, Huazhi Rougan granule, Qiaozhi capsule, Liuwei Wuling tablet, Hedan tablet, Gandan Shukang capsule, Xuezhikang capsule, Yiganling tablet, Hugan capsule, Zhibitai capsule). The network relationship between the interventions is shown in Figure 3. In terms of TC improvement, according to MD and 95%CI between all the pairwise interventions, Dangfei Liganning capsule (MD: 1.08; 95%CI: 0.67, 1.49), Liuwei Wuling tablet (MD: 1.04; 95%CI: 0.17, 1.91), Yiganling tablet (MD: 0.99; 95%CI: 0.09, 1.89) were superior to placebo. Zhibitai capsule (MD: 0.71; 95%CI: 0.32, 1.10) was superior to lifestyle modification. Dangfei Liganning capsule (MD: 1.90; 95%CI: 1.25, 2.55), Huazhi Rougan granule (MD: 0.77; 95%CI: 0.57, 0.98), Qiaozhi capsule (MD: 0.43; 95%CI: 0.15, 0.70), Liuwei Wuling tablet (MD: 1.86; 95%CI: 1.45, 2.27), Hedan tablet (MD: 0.46; 95%CI: 0.10, 0.83) and Yiganling tablet (MD: 1.81; 95%CI: 0.78, 2.84) were superior to western medicine. In addition, Dangfei Liganning capsule was superior to Qianggan capsule (MD: −1.59; 95%CI: −2.31, −0.87), Danning tablet (MD: 1.65; 95%CI: 0.93, 2.38), Huazhi Rougan granule (MD: 1.13; 95%CI: 0.45, 1.81), Qiaozhi capsule (MD: 1.47; 95%CI: 0.77, 2.18), Hedan tablet (MD: 1.44; 95%CI: 0.69, 2.18), Gandan Shukang capsule (MD: 1.42; 95%CI: 0.62, 2.22), Xuezhikang capsule (MD: 1.77; 95%CI: 1.02, 2.52), Hugan capsule (MD: 1.40; 95%CI: 0.98, 1.82). Liuwei Wuling tablet was superior to Qianggan capsule (MD: −1.55; 95%CI: −2.07, −1.03), Danning tablet (MD: −1.61; 95%CI: −2.14, −1.08), Huazhi Rougan granule (MD: −1.09; 95%CI: −1.55, −0.63), Qiaozhi capsule (MD: −1.43; 95%CI: −1.93, −0.94), Hedan tablet (MD: 1.40; 95%CI: 0.84, 1.95), Gandan Shukang capsule (MD: 1.38; 95%CI: 0.75, 2.01), Xuezhikang capsule (MD: 1.73; 95%CI: 1.17, 2.29), Hugan capsule (MD: 1.36; 95%CI: 0.49, 2.23). Yiganling tablet was superior to Qianggan capsule (MD: −1.50; 95%CI: −2.58, −0.42), Danning tablet (MD: −1.56; 95%CI: −2.64, −0.48), Qiaozhi capsule (MD: −1.38; 95%CI: −2.45, −0.32), Hedan tablet (MD: −1.35; 95%CI: −2.44, −0.25), Gandan Shukang capsule (MD: −1.33; 95%CI: −2.47, −0.19), Xuezhikang capsule (MD: −1.68; 95%CI: −2.78, −0.58), Hugan capsule (MD: 1.31; 95%CI: 0.40, 2.22). Huazhi Rougan granule was superior to Qianggan capsule (MD: −0.46; 95%CI: −0.84, −0.08), Danning tablet (MD: −0.52; 95%CI: −0.91, −0.14), Xuezhikang capsule (MD: 0.64; 95%CI: 0.21, 1.08). The detailed results of pairwise comparison are shown in Table 4 and Supplementary Figure S6. Moreover, Dangfei Liganning capsule, with the highest-ranking probability of SUCRA (91.1%), had the best effectiveness in reducing TC, followed by Liuwei Wuling tablet (90.5%) and Yiganling tablet (87.9%). More details about the rank probability of SUCRA are shown in Figure 4.

### 7.5 Adverse reactions

22 studies mentioned adverse reactions. 12 studies reported that neither group had adverse reactions. The other 10 studies reported specific adverse reactions, including Liuwei Wuling tablet and Gandan Shukang capsule. Adverse reactions mainly occurred in Qianggan capsule, Danning tablet, Huazhi Rougan granule, Qiaozhi capsule, Hedan tablet and Xuezhikang capsule, but most of them were mild gastrointestinal reactions. Specific adverse reactions are shown in Supplementary Table 10.

### 7.6 Publication bias

**FIGURE 5:**
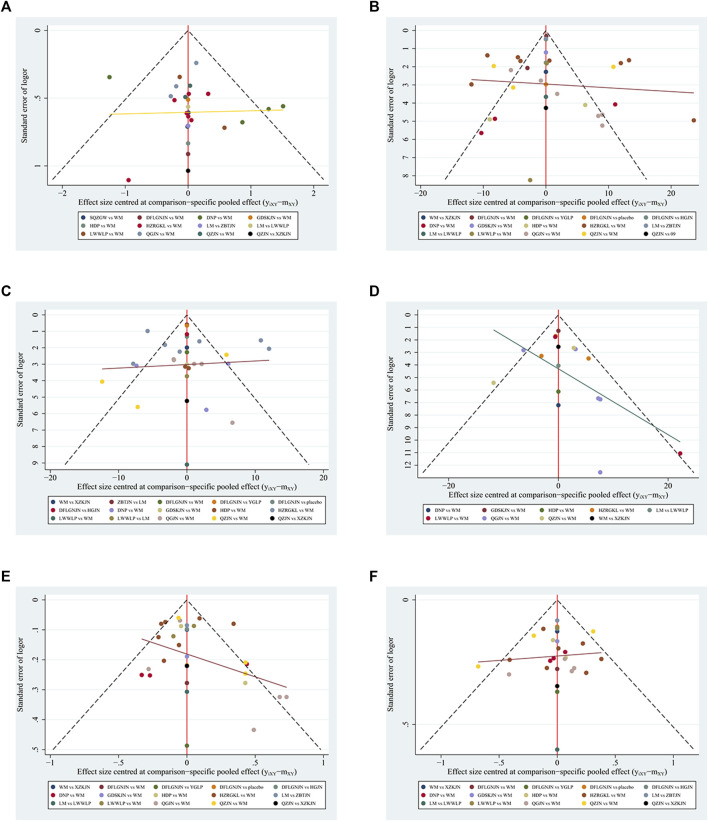
Funnel plots **(A)** Clinical efficiency rate **(B)** ALT **(C)** AST **(D)** GGT **(E)** TG **(F)** TC; WM, Western medicine; QGJN, Qianggan capsule; DFLGNJN, Dangfei Liganning capsule; DNP, Danning tablets; HZRGKL, Huazhi Rougan granule; QZJN, Qiaozhi capsule; SQZGW, Sanqi Zhigan pill; LWWLP, Liuwei Wuling tablet; HDP, Hedan tablet; GDSKJN, Gandan Shukang capsule; XZKJN, Xuezhikang capsule; YGLP, Yiganling tablet; HGJN, Hugan capsule; ZBTJN, Zhibitai capsule; LM, lifestyle modification.

The comparison adjusted funnel plot for the six outcomes is shown in Figure 5. The comparison adjusted funnel chart for the outcome indicator of clinical efficiency rate showed that its symmetry was poor, and there might be publication bias. The reason may be related to the small number of included studies and the small total sample size. The comparison adjusted funnel chart for the five outcome indicators ALT, AST, GGT, TG and TC showed that all studies were symmetrically distributed in the upper middle part and clustered towards the middle line, suggesting that the risk of publication bias were low.

### 7.7 Network inconsistency and heterogeneity

The network evidence diagrams of ALT, AST, TG and TC formed a closed loop respectively. Node splitting analysis was used to evaluate inconsistency. The NMA did not show any inconsistency in ALT, AST, TG and TC. The details are shown in Supplementary Figures S7–S10. As shown in Supplementary Table 11, the global *I* ^*2*^ of clinical efficiency rate and ALT were54.2%, 94.2% respectively. The global *I* ^*2*^ of AST, GGT, TG, TC were 91.1%, 73.6%, 80.3%, 44.2% respectively. Sensitivity analysis showed that excluding any one study would not change the expected confidence interval, and the pooled results were stable. The results of sensitivity analysis are shown in Supplementary Figures S11–S13.

## 8 Discussion

Traditional Chinese medicine (TCM) believes that the causes of NAFLD patients are eating much fat and sweet, lying more and moving less, and emotional disorders. These causes lead to liver loss, spleen loss, endogenous dampness, phlegm accumulation, kidney essence loss, and phlegm retention. Finally, it will cause dysfunction of the liver, spleen and kidney. Phlegm, dampness and stasis will block the liver collaterals (Zhang and Li, 2017). In clinical practice, the main treatment principle should be “resolving phlegm, removing dampness and activating blood circulation”. All CPMs included in these studies are Chinese herbal prescriptions established on the basis of the above treatment principles.

Based on 39 related research materials and six main results, we systematically evaluated the efficacy of 13 commonly used CPMs in the treatment of NAFLD by using a network meta-analysis technique. According to the results of NMA, most CPMs were superior to placebo or lifestyle modification or western medicine in all results. The difference between groups was statistically significant. According to the results of NMA, except for AST, most CPMs were better than placebo or lifestyle modification or western medicine in the outcomes, with statistically significant differences between groups. The reason why there was no significant difference in serum AST may be that the patients with NAFLD often showed an increase in serum ALT and GGT, while the increase in serum AST was not significant. Therefore, there was no significant difference between the two comparisons in this NMA.

As far as the results of this NMA were concerned, Zhibitai capsule had the best efficacy in improving clinical efficiency rate. It is composed of hawthorn, rhizoma alismatis, atractylodes macrocephala and monascus, which has the effect of eliminating phlegm, dampness and blood stasis. It directly treats the phlegm, dampness and blood stasis of NAFLD core pathogenesis. Therefore, it can significantly improve the clinical efficacy. In addition, modern research showed that maslinic acid in hawthorn could reduce the content of fat in the liver cells of NAFLD mice and inflammation injury (Li et al., 2022). Alisol B in alisma orientalis could attenuate hepatic steatosis, inflammation, and fibrosis in high-fat diet plus carbon tetrachloride (DIO + CCl4)-induced and choline-deficient and amino acid-defined (CDA)-diet-induced NASH mice (Zhao Z et al., 2022). Monascus had the effects of lowering blood lipid and anti-inflammation (Hsu et al., 2014). Liuwei Wuling tablet had the best effect in reducing serum ALT and AST level for NAFLD patients. It is composed of *Schisandra chinensis*, *Ligustrum lucidum*, *Forsythia suspensa*, Curcuma zedoary, *Sonchus* chicory, Ganoderma lucidum spore powder, which has the effect of promoting blood circulation and nourishing liver. Long term stagnation of blood stasis may lead to liver inflammation. Liuwei Wuling tablet has the effect of promoting blood circulation and nourishing liver. Therefore, it is more effective in improving the inflammatory damage of NAFLD. Modern pharmacological studies showed that the extract of *Schisandra chinensis* could reduce liver damage and serum ALT, AST levels (Zhao J et al., 2022). Liqustri lucidi Fructus could regulate AMPK signaling pathway to protect hepatocytes from oxidative damage (Seo et al., 2017). Phillygenin, an extract of *Forsythia suspensa*, could reduce liver lipid deposition and serum ALT, AST levels in NAFLD mice induced by high-fat diet (Zhou W et al., 2022). Ganoderma lucidum spore powder could protect mice against developing obesity caused by increased fat intake by regulating inflammatory factors and lipid metabolism (Zhong et al., 2022). Gandan Shukang capsule had the best effect in reducing serum GGT level. It is composed of white peony, herba artemisiae, bupleurum, turmeric, salvia miltiorrhiza, turtle shell (made of) and jujube. It has the effect of clearing the liver, regulating the spleen, promoting qi and removing blood stasis. The effect of Gandan Shukang capsule on removing blood stasis might be the reason for its improvement of NAFLD inflammation. Paeoniflorin, the extract of paeony, could improve the liver inflammation and reduce serum GGT level in rats with non-alcoholic steatohepatitis (Ma et al., 2016; Ma et al., 2020). Saikosaponin-d, the extract of Bupleurum chinense, could inhibit the inflammatory reaction in NAFLD mice and achieve the liver protective effect (Chang et al., 2021). Salvianolic acid B, Tanshinone IIA and Salvianolic acid A in Salvia miltiorrhiza extract could improve the inflammatory damage in NAFLD mice (Li et al., 2020; Meng et al., 2022; Xu et al., 2022). Qianggan capsule had the best effect in reducing serum TG level. It is composed of Herba Artemisiae, Radix Isatidis, Angelica, Radix Paeoniae Alba, Radix Salviae Miltiorrhizae, Radix Curcumae, Radix Astragali, Codonopsis, Rhizoma Alisma, Rhizoma Polygonati, Rhizoma Rehmanniae, Chinese Yam, Hawthorn, Six God Qu, *Gentiana macrophylla*, and *Glycyrrhiza*. It has the effects of clearing away heat and dampness, nourishing the spleen and blood, and supplementing qi and relieving depression. Its diuretic effect may be the reason why it reduces blood lipids. Diosgenin is abundant in yam, Study confirmed that the abundant diosgenin in yam could suppress excessive weight gain, reduce serum levels of total cholesterol and triglycerides, and decrease liver fat accumulation in high-fat diet-induced NAFLD rats (Zhou Y et al., 2022). Hawthorn could reduce blood lipid, especially serum TG level (Al Humayed, 2016; Han et al., 2016; Kim et al., 2022). Curcumin in turmeric could reduce blood lipid (Jarhahzadeh et al., 2021). Dangfei Liganning capsule had the best effect in reducing serum TC level. It is composed of local medicine and silymarin, which has the effect of clearing damp heat, benefiting liver and removing jaundice. Its diuretic effect could also reduce blood lipids. Modern pharmacological research showed that sweroside may ameliorate obesity with fatty liver *via* the regulation of lipid metabolism and inflammatory responses (Yang et al., 2020). Silymarin is a mixture of flavonoid obtained from *Silybum marianum* (L.) Gaertn. [*Asteraceae*] with good clincial evidence for liver protecting effect and a wide use in phytotherapy especially in Europe. It can also reduce blood lipids. Many studies had shown that silymarin, an extract of silymarin, could regulate lipid metabolism, reduce blood lipids, and achieve the goal of treating NAFLD (Ni and Wang, 2016; Jiang et al., 2022). Some scholars also pointed out that silymarin could be used for NAFLD without elevated transaminase (Liu et al., 2022). None of the included studies had serious adverse reactions. Most of the studies only showed mild gastrointestinal reactions, which could be eliminated after relevant treatment.

The complete mechanism of CPMs in treating NAFLD is still unclear. Some potential mechanisms have been clarified. Animal experiments showed that Qianggan capsule had a good therapeutic effect on liver lipid and inflammation in NAFLD model of SD male rats prepared with high-fat diet. The possible mechanism was to improve leptin resistance and increased the expression of leptin receptor mRNA and phosphorylated protein tyrosine kinase JAK-2 (P-JAK2), signal transducer and activator of transcription three phosphorylation (P-STAT3) protein in the liver (Zheng et al., 2009). Another study showed that Qianggan capsule might improve the NAFLD model of Wistar rats prepared with high-fat diet by inhibiting the expression of interleukin-8 (IL-8) mediated by early growth response protein 1 (EGR-1) (Hao and Liu, 2018). The mechanism of Dangfei Liganning capsule in treating NAFLD might be to reduce subcellular localization of nuclear factor E2-related factor 2 (Nrf2) mediated liver oxidative stress, reduce the expression of NOD-like receptor thermal protein domain associated protein 3 (NLRP3) inflammatory body related genes and nuclear factor kappa-B (NF-ΚB) protein (Xu et al., 2015; Zhao et al., 2021). In addition, the regulation of Dangfei Liganning capsule on adiponectin, tumor necrosis factor-α (TNF-α) and insulin resistance was also an important mechanism for its treatment of NAFLD (Song et al., 2012). The mechanism of Danning tablet in the treatment of NAFLD was to reduce the content of TNF-α, transforming growth factor-β1 (TGF-β1), malondialdehyde (MDA) in liver tissue and increase the content of succinate dehydrogenase (SDH) (Zhang et al., 2016; Jiang et al., 2017). Huazhi Rougan granule could prevent NAFLD by reducing the levels of cytokines interleukin18 (IL-18) and interleukin-1β (IL-1β) (Shi et al., 2020). Qiaozhi capsule could regulate the secretion of GLUT-4, and significantly reduce the secretion of TNF-α and interleukin6 (IL-6). It could also improve IR and regulate fat metabolism by up regulating the protein and gene expression of peroxisome proliferator-activated receptor γ (PPAR-γ) and IR in liver tissue. In addition, it could reduce the expression of heme oxygenase 1 (HO-1) and cytochrome P450 family member 2E1 (CYP2E1) in liver tissue, reduce oxidative stress and lipid peroxidation, and prevent the occurrence and development of NAFLD (Wang et al., 2011; Zhao et al., 2014; Zhao et al., 2015).

This study still has the following limitations: ① The quality of the included studies was low. In the 39 studies, only 19 studies reported random methods, and one study used a double-blind method. Most of the literatures quality was evaluated as “unclear”, which led to a certain risk of publication bias; ② The included studies were all small sample studies, which would reduce the statistical reliability of this study; ③ Not all the CPMs included in these studies could follow the principle of “syndrome differentiation and treatment”. There were unified interventions for NAFLD of different syndrome types, which might affect the results; ④ The length of treatment varied. Most treatment cycles were 12 weeks and 24 weeks, but a few studies had treatment cycles of 8 weeks, which might cause clinical heterogeneity. ⑤ The number of RCTs involved in Zhibitai Capsule (2RCTs), Liuwei Wuling Tablet (3RCTs), Gandan Shukang Capsule (1RCT), Dangfei Liganning Capsule (5RCTs), Qianggan Capsule (6RCTs) is limited, and the results of NMA merger may not be convincing enough. ⑥ All these studies were conducted in China, which has certain restrictions on the promotion and application of Chinese patent medicines. In view of the above limitations, it is suggested that the following four points should be noted during the implementation of future studies: ① Apply the correct randomization method. For example, a random number table or a computer is used to generate random numbers. At the same time, attention should be paid to hiding the random serial number, such as using orderly numbered, opaque and sealed envelopes to hide the random number. These can avoid selectivity bias. ② The study design shall be at least double blind. The blind method shall be adopted for both the subjects and the main researchers, and the blind method shall not be easily damaged to avoid implementation bias. ③ It is recommended that all RCT studies should be registered for clinical trials before implementation, so as to avoid publication bias due to only reporting positive outcomes.④ It is suggested to carry out multi center, large sample studies of international cooperation to clarify the exact efficacy of these Chinese patent medicines in the treatment of NAFLD.

## 9 Conclusion

For patients with NAFLD in this NMA, Zhibitai capsule might have best efficacy in improving clinical efficiency rate, Liuwei Wuling tablet might have best effect in reducing serum ALT and AST level, Gandan Shukang capsule might have best effect in reducing serum GGT level, Qianggan capsule might have best effect in reducing serum TG level, Dangfei Liganning capsule might have best effect in reducing serum TC level. The results of this NMA are limited to the data analysis of literatures, and cannot fully explain the clinical efficacy of CPMs in treating NAFLD, so there are some limitations. Better designed double-blind, multi center and large sample RCTs are needed which resolve the problems of blinding, selective reporting and allocation concealment.

## Data Availability

The original contributions presented in the study are included in the article/Supplementary Material; further inquiries can be directed to the corresponding authors.

## Glossary

ALT: Alanine aminotransferase
AST: Aspartate aminotransferase
CPMs: Chinese patent medicines
CPM: Chinese patent medicine
CT: Computed tomography
CNKI: China National Knowledge Infrastructure
CYP2E1: Cytochrome P450 family member 2E1
CI: Confidence interval
CBM: Chinese Biomedical Literature Database
DFLGNJN: Dangfei Liganning capsule
DNP: Danning tablet
GDSKJN: Gandan Shukang capsule
GGT: Gamma glutamyl transferase
HZRGKL: Huazhi Rougan granule
HDP: Hedan tablet
HO-1: Heme oxygenase 1
IL-18: Interleukin18
IL-1β: Interleukin-1β
IR: Insulin resistance
LDL: Low density lipoprotein
LM: lifestyle modification
LWWLP: Liuwei Wuling tablet
MAFLD: Metabolic associated fatty liver disease
MASH: Metabolic associated steatohepatitis
MD: Mean differences
MDA: Malondialdehyde
NMA: Network meta-analysis
NAFLD: Non-alcoholic fatty liver disease
NASH: Non-alcoholic steatohepatitis
Nrf2: Nuclear factor E2-related factor 2
NLRP3: NOD-like receptor thermal protein domain associated protein 3
NF-ΚB: Nuclear factor kappa-B
ORs: Odds ratios
PPAR-γ: peroxisome proliferator-activated receptor γ
QGJN: Qianggan capsule
QZJN: Qiaozhi capsule
RCTs: Randomized clinical trials
SDH: Succinate dehydrogenase
SQZGW: Sanqi Zhigan pill
SUCRA: Surface Under the Cumulative Ranking
TG: Triglyceride
TC: Total cholesterol
TNF-α: Tumor necrosis factor-α
TGF-β1: Transforming growth factor-β1
VIP: China Science and Technology Journal Database
WM: Western medicine
XZKJN: Xuezhikang capsule
YGLP: Yiganling tablet
HGJN: Hugan capsule
ZBTJN: Zhibitai capsule
