# Supplementary material for: Comparative efficacy of Chinese patent medicines for non-alcoholic fatty liver disease: A network meta-analysis
Source: Front Pharmacol. 2023 Jan 4;13:1077180. doi: 10.3389/fphar.2022.1077180 (PMC9847677; doi:10.3389/fphar.2022.1077180)
Supplement: Supplementary file 1 [file DataSheet1.pdf]

Supplementary Material

1 PRISMA guideline *2*

2 Search strategy *8*

3 Details of the included CPMs *13*

4 Forest plots *19*

5 Adverse reactions *25*

6 Inconsistency plots *29*

7 Heterogeneity analysis *31*

8 Sensitivity analysis *32*

1 PRISMA guideline

Supplementary Table 1 PRISMA checklist of the network meta-analysis

Section and Topic	Item #	Checklist item	Location where item is reported
**TITLE**			
Title	1	Identify the report as a systematic review.	1
**ABSTRACT**			
Abstract	2	See the PRISMA 2020 for Abstracts checklist.	1-2
**INTRODUCTION**			
Rationale	3	Describe the rationale for the review in the context of existing knowledge.	2
Objectives	4	Provide an explicit statement of the objective(s) or question(s) the review addresses.	2
**METHODS**			
Eligibility criteria	5	Specify the inclusion and exclusion criteria for the review and how studies were grouped for the syntheses.	3
Information	6	Specify all databases, registers, websites, organisations, reference lists and other sources searched or consulted to	3

Section and Topic	Item #	Checklist item	Location where item is reported
sources		identify studies. Specify the date when each source was last searched or consulted.	
Search strategy	7	Present the full search strategies for all databases, registers and websites, including any filters and limits used.	3
Selection process	8	Specify the methods used to decide whether a study met the inclusion criteria of the review, including how many reviewers screened each record and each report retrieved, whether they worked independently, and if applicable, details of automation tools used in the process.	4
Data collection process	9	Specify the methods used to collect data from reports, including how many reviewers collected data from each report, whether they worked independently, any processes for obtaining or confirming data from study investigators, and if applicable, details of automation tools used in the process.	4
Data items	10a	List and define all outcomes for which data were sought. Specify whether all results that were compatible with each outcome domain in each study were sought (e.g. for all measures, time points, analyses), and if not, the methods used to decide which results to collect.	4
	10b	List and define all other variables for which data were sought (e.g. participant and intervention characteristics, funding sources). Describe any assumptions made about any missing or unclear information.	4-5
Study risk of bias	11	Specify the methods used to assess risk of bias in the included studies, including details of the tool(s) used, how many reviewers assessed each study and whether they worked independently, and if applicable, details of automation tools	3-4

Section and Topic	Item #	Checklist item	Location where item is reported
assessment		used in the process.	
Effect measures	12	Specify for each outcome the effect measure(s) (e.g. risk ratio, mean difference) used in the synthesis or presentation of results.	4
Synthesis methods	13a	Describe the processes used to decide which studies were eligible for each synthesis (e.g. tabulating the study intervention characteristics and comparing against the planned groups for each synthesis (item #5)).	3
	13b	Describe any methods required to prepare the data for presentation or synthesis, such as handling of missing summary statistics, or data conversions.	4
	13c	Describe any methods used to tabulate or visually display results of individual studies and syntheses.	4
	13d	Describe any methods used to synthesize results and provide a rationale for the choice(s). If meta-analysis was performed, describe the model(s), method(s) to identify the presence and extent of statistical heterogeneity, and software package(s) used.	4
	13e	Describe any methods used to explore possible causes of heterogeneity among study results (e.g. subgroup analysis, meta-regression).	4
	13f	Describe any sensitivity analyses conducted to assess robustness of the synthesized results.	4
Reporting bias	14	Describe any methods used to assess risk of bias due to missing results in a synthesis (arising from reporting biases).	3-4

Section and Topic	Item #	Checklist item	Location where item is reported
assessment			
Certainty assessment	15	Describe any methods used to assess certainty (or confidence) in the body of evidence for an outcome.	4
**RESULTS**			
Study selection	16a	Describe the results of the search and selection process, from the number of records identified in the search to the number of studies included in the review, ideally using a flow diagram.	4-5
	16b	Cite studies that might appear to meet the inclusion criteria, but which were excluded, and explain why they were excluded.	4-5
Study characteristics	17	Cite each included study and present its characteristics.	4-5
Risk of bias in studies	18	Present assessments of risk of bias for each included study.	5
Results of individual studies	19	For all outcomes, present, for each study: (a) summary statistics for each group (where appropriate) and (b) an effect estimate and its precision (e.g. confidence/credible interval), ideally using structured tables or plots.	5-8

Section and Topic	Item #	Checklist item	Location where item is reported
Results of syntheses	20a	For each synthesis, briefly summarise the characteristics and risk of bias among contributing studies.	5-8
	20b	Present results of all statistical syntheses conducted. If meta-analysis was done, present for each the summary estimate and its precision (e.g. confidence/credible interval) and measures of statistical heterogeneity. If comparing groups, describe the direction of the effect.	5-8
	20c	Present results of all investigations of possible causes of heterogeneity among study results.	8
	20d	Present results of all sensitivity analyses conducted to assess the robustness of the synthesized results.	8
Reporting biases	21	Present assessments of risk of bias due to missing results (arising from reporting biases) for each synthesis assessed.	5
Certainty of evidence	22	Present assessments of certainty (or confidence) in the body of evidence for each outcome assessed.	5
**DISCUSSION**			
Discussion	23a	Provide a general interpretation of the results in the context of other evidence.	9-10
	23b	Discuss any limitations of the evidence included in the review.	9-10
	23c	Discuss any limitations of the review processes used.	9-10

Section and Topic	Item #	Checklist item	Location where item is reported
	23d	Discuss implications of the results for practice, policy, and future research.	9-10
**OTHER INFORMATION**			
Registration and protocol	24a	Provide registration information for the review, including register name and registration number, or state that the review was not registered.	2
	24b	Indicate where the review protocol can be accessed, or state that a protocol was not prepared.	2
	24c	Describe and explain any amendments to information provided at registration or in the protocol.	2
Support	25	Describe sources of financial or non-financial support for the review, and the role of the funders or sponsors in the review.	11
Competing interests	26	Declare any competing interests of review authors.	11
Availability of data, code and other materials	27	Report which of the following are publicly available and where they can be found: template data collection forms; data extracted from included studies; data used for all analyses; analytic code; any other materials used in the review.	10

2 Search strategy

Supplementary Table 2 Search strategy in Pubmed

No.	Search items
#1	Non-alcoholic Fatty Liver Disease [MeSH Terms]
#2	Non alcoholic Fatty Liver Disease[Title/Abstract] OR NAFLD[Title/Abstract] OR Nonalcoholic Fatty Liver Disease[Title/Abstract] OR Fatty Liver, Nonalcoholic[Title/Abstract] OR Fatty Livers, Nonalcoholic[Title/Abstract] OR Liver, Nonalcoholic Fatty[Title/Abstract] OR Livers, Nonalcoholic Fatty[Title/Abstract] OR Nonalcoholic Fatty Liver[Title/Abstract] OR Nonalcoholic Fatty Livers[Title/Abstract] OR Nonalcoholic Steatohepatitis[Title/Abstract] OR Steatohepatitides, Nonalcoholic[Title/Abstract] OR Steatohepatitis, Nonalcoholic[Title/Abstract] OR NASH[Title/Abstract] OR non-alcoholic steatohepatitis[Title/Abstract] OR metabolic associated fatty liver disease[Title/Abstract] OR metabolic associated steatohepatitis[Title/Abstract] OR MAFLD[Title/Abstract] OR MASH[Title/Abstract]
#3	#1 OR #2
#4	Proprietary Chinese medicine[Title/Abstract] OR Chinese patent medicine[Title/Abstract] OR capsule[Title/Abstract] OR tablet[Title/Abstract] OR pellet[Title/Abstract] OR pill[Title/Abstract] OR powder[Title/Abstract]
#5	randomized controlled trial[Publication Type] OR randomized[Title/Abstract] OR placebo[Title/Abstract]
#6	#3 AND #4 AND #5

Supplementary Table 3 Search strategy in Embase

No.	Search items
#1	'nonalcoholic fatty liver'/exp
#2	"Non-alcoholic Fatty Liver Disease":ti,ab OR "Non alcoholic Fatty Liver Disease":ti,ab OR "NAFLD":ti,ab OR "Nonalcoholic Fatty Liver Disease":ti,ab OR "Fatty Liver, Nonalcoholic":ti,ab OR "Fatty Livers, Nonalcoholic":ti,ab OR "Liver, Nonalcoholic Fatty":ti,ab OR "Livers, Nonalcoholic Fatty":ti,ab OR

	"Nonalcoholic Fatty Liver":ti,ab OR "Nonalcoholic Fatty Livers":ti,ab OR "Nonalcoholic Steatohepatitis":ti,ab OR "Steatohepatitides, Nonalcoholic":ti,ab OR "Steatohepatitis, Nonalcoholic":ti,ab OR "NASH":ti,ab OR "non-alcoholic steatohepatitis":ti,ab OR "metabolic associated fatty liver disease":ti,ab OR "metabolic associated steatohepatitis":ti,ab OR "MAFLD":ti,ab OR "MASH":ti,ab
#3	#1 OR #2
#4	"Proprietary Chinese medicine":ti,ab OR "Chinese patent medicine":ti,ab OR "capsule":ti,ab OR "tablet":ti,ab OR "pellet":ti,ab OR "pill":ti,ab OR "powder":ti,ab
#5	"random":ti,ab OR "placebo":ti,ab OR "double-blind":ti,ab
#6	#3 AND #4 AND #5

Supplementary Table 4 Search strategy in Cochrane Library

No.	Search items
#1	MeSH descriptor: [Non-alcoholic Fatty Liver Disease] explode all trees
#2	(Non alcoholic Fatty Liver Disease):ti,ab OR (NAFLD):ti,ab OR (Nonalcoholic Fatty Liver Disease):ti,ab OR (Fatty Liver, Nonalcoholic):ti,ab OR (Fatty Livers, Nonalcoholic):ti,ab OR (Liver, Nonalcoholic Fatty):ti,ab OR (Livers, Nonalcoholic Fatty):ti,ab OR (Nonalcoholic Fatty Liver):ti,ab OR (Nonalcoholic Fatty Livers):ti,ab OR (Nonalcoholic Steatohepatitis):ti,ab OR (Steatohepatitides, Nonalcoholic):ti,ab OR (Steatohepatitis, Nonalcoholic):ti,ab OR (NASH):ti,ab OR (non-alcoholic steatohepatitis):ti,ab OR (metabolic associated fatty liver disease):ti,ab OR (metabolic associated steatohepatitis):ti,ab OR (MAFLD):ti,ab OR (MASH):ti,ab
#3	#1 OR #2
#4	(Proprietary Chinese medicine):ti,ab OR (Chinese patent medicine):ti,ab OR (capsule):ti,ab OR (tablet):ti,ab OR (pellet):ti,ab OR (pill):ti,ab OR (powder):ti,ab
#5	3 AND #4

Supplementary Table 5 Search strategy in Web of Science

No.	Search items
#1	TS=(Non-alcoholic Fatty Liver Disease) OR TS=(Non alcoholic Fatty Liver Disease) OR TS=(NAFLD) OR TS=(Nonalcoholic Fatty Liver Disease) OR TS=(Fatty Liver, Nonalcoholic) OR TS=(Fatty Livers, Nonalcoholic) OR TS=(Liver, Nonalcoholic Fatty OR Livers, Nonalcoholic Fatty) OR TS=(Nonalcoholic Fatty Liver) OR TS=(Nonalcoholic Fatty Livers) OR TS=(Nonalcoholic Steatohepatitis) OR TS=(steatohepatities, Nonalcoholic) OR TS=(Steatohepatitis, Nonalcoholic) OR TS=(NASH) OR TS=(non-alcoholic steatohepatitis) OR TS=(metabolic associated fatty liver disease) OR TS=(metabolic associated steatohepatitis) OR TS=(mafsd) OR TS=(MASH)
#2	TS=(Proprietary Chinese medicine) OR TS=(Chinese patent medicine) OR TS=(capsule) OR TS=(tablet) OR TS=(pellet OR pill OR powder)
#3	TS=(Random* controlled trial) OR TS=(random*) OR TS=(placebo)
#4	#1 AND #2 AND #3

Supplementary Table 6 Search strategy in China National Knowledge Infrastructure

No.	Search items
#1	SU %= '非酒精性脂肪性肝病' OR SU %= '非酒精性脂肪肝' OR SU %= '非酒精性脂肪肝病' OR SU %= '非酒精性单纯性脂肪肝' OR SU %= '非酒精性脂肪性肝炎' OR SU %= '代谢相关脂肪性肝病' OR SU %= '代谢相关脂肪性肝炎' OR SU %= 'NAFLD' OR SU %= 'NASH' OR SU %= 'MAFLD' OR SU %= 'MASH'
#2	SU %= '中成药' OR SU %= '胶囊' OR SU %= '片' OR SU %= '颗粒' OR SU %= '丸' OR SU %= '散'
#3	FT = '随机'
#4	#1 AND #2 AND #3

Supplementary Table 7 Search strategy in Wanfang Database

No.	Search items

#1	主题:(非酒精性脂肪性肝病) or 主题:(非酒精性脂肪肝) or 主题:(非酒精性脂肪肝病) or 主题:(非酒精性单纯性脂肪肝) or 主题:(非酒精性脂肪性肝炎)or 主题:(代谢相关脂肪性肝病) or 主题:(代谢相关脂肪性肝炎) or 主题:(NAFLD) or 主题:(NASH) or 主题:(MAFLD) or 主题:(MASH)
#2	主题:(中成药) or 主题:(胶囊) or 主题:(片) or 主题:(颗粒) or 主题:(丸) or 主题:(散)
#3	摘要:(随机)
#4	#1 AND #2 AND #3

Supplementary Table 8 Search strategy in China Science and Technology Journal Database

No.	Search items
#1	M=(非酒精性脂肪性肝病 OR 非酒精性脂肪肝 OR 非酒精性脂肪肝病 OR 非酒精性单纯性脂肪肝 OR 非酒精性脂肪性肝炎 OR 代谢相关脂肪性肝病 OR 代谢相关脂肪性肝炎 OR NAFLD OR NASH OR MAFLD OR MASH)
#2	M=(中成药 OR 胶囊 OR 片 OR 颗粒 OR 丸 OR 散)
#3	U=(随机)
#4	#1 AND #2 AND #3

Supplementary Table 9 Search strategy in Chinese Biomedical Literature Database.

No.	Search items
#1	"非酒精性脂肪性肝病"[常用字段:智能] OR "非酒精性脂肪肝"[常用字段:智能] OR "非酒精性脂肪肝病"[常用字段:智能] OR "非酒精性单纯性脂肪肝"[常用字段:智能] OR "非酒精性脂肪性肝炎"[常用字段:智能] OR "代谢相关脂肪性肝病"[常用字段:智能] OR "代谢相关脂肪性肝炎"[常用字段:智能] OR "NAFLD"[常用字段:智能] OR "NASH"[常用字段:智能] OR "MAFLD"[常用字段:智能] OR "MASH"[常用字段:智能]

#2	"中成药"[常用字段:智能] OR "胶囊"[常用字段:智能] OR "片"[常用字段:智能] OR "颗粒"[常用字段:智能] OR "丸"[常用字段:智能] OR "散"[常用字段:智能]
#3	"随机"[常用字段:智能])
#4	#1 AND #2 AND #3

3 Details of the included CPMs

Supplementary Table 10 Details of the included CPMs.

CPMs	Source	SFDA approval number	Scientific name of Plant or Animal	Quality control reported? (Y/N)	Chemical analysis reported? (Y/N)
Qianggan capsule	Shijiazhuang Dongfang Pharmaceutical Co. Ltd.	Z10980012	*Artemisia capillaris* Thunb. [Asteraceae; Artemisiae scopariae herba] (Yinchen); *Isatis tinctoria* L. [Brassicaceae; Isatidis radix] (Banlangen);*Angelica sinensis* (Oliv.) Diels [Apiaceae; Angelicae sinensis radix] (Danggui);*Paeonia lactiflora* Pall. [Paeoniaceae; Paeoniae radix alba] (Baishao);*Salvia miltiorrhiza* Bunge [Lamiaceae; Salviae miltiorrhizae radix et rhizoma] (Danshen);*Curcuma longa* L. [Zingiberaceae; Curcumae radix] (Yujin);*Astragalus mongholicus* Bunge [Fabaceae; Astragali radix] (Huangqi);*Codonopsis pilosula* (Franch.) Nannf. [Campanulaceae; Codonopsis radix] (Dangshen);*Alisma plantago-aquatica subsp. orientale* (Sam.) Sam. [Alismataceae; Alismatis rhizoma] (Zexie);*Polygonatum cyrtonema* Hua [Asparagaceae; Polygonati rhizoma] (Huangjing);*Rehmannia glutinosa* (Gaertn.) DC. [Orobanchaceae; Rehmanniae radix] (Dihuang);*Dioscorea opposita* Thunb. [Dioscoreaceae; Dioscoreae rhizoma] (Shanyao);*Crataegus pinnatifida* Bunge [Rosaceae; Crataegi fructus] (Shanzha);Massa medicata fermentata [Composed of Polygonum hydropiper, Artemisia annua, Xanthium sibiricum, red bean, bitter almond, wheat bran and flour] (Liushenqu); *Gentiana macrophylla* Pall. [Gentianaceae; Gentianae macrophyllae radix] (Qinjiao); *Glycyrrhiza glabra* L. [Fabaceae; Glycyrrhizae radix et rhizoma] (Gancao)<br><br>Unknown dosage	Y*	Y-HPLC (Wei et al., 2013), HPLC (Ma et al., 2012)
Dangfei Ligannin g capsule	Sichuan Meidakang Pharmaceutic	Z51020085	*Silybum marianum* (L.) Gaertn. [Asteraceae; Silybi fructus] (Shuifeiji), 900g; *Swertia pseudochinensis* H.Hara [Gentianaceae; Swertiae herba] (Dangyao), 950g;	Y*	Y-HPLC (Pan et al., 2018), HPLC (Long

	al Co. Ltd.		Made into 1,000 capsules		et al., 2017)
Danning tablet	Amendment Pharmaceutical Group Co. Ltd.	Z22020983	*Rheum palmatum* L. [Polygonaceae; Rhei radix et rhizoma] (Dahuang), 48g; *Reynoutria japonica* Houtt. [Polygonaceae; Polygoni cuspidati rhizoma et radix] (Huzhang), 720g; *Citrus × aurantium* L. [Rutaceae; Citri reticulatae pericarpium viride] (Qingpi), 288g; *Imperata cylindrica* (L.) P.Beauv. [Poaceae; Imperatae rhizoma] (Baimaogen), 432g; *Citrus × aurantium* L. [Rutaceae; Citri reticulatae pericarpium] (Chenpi), 288g; *Curcuma longa* L. [Zingiberaceae; Curcumae radix] (Yujin), 432g; *Crataegus pinnatifida* Bunge [Rosaceae; Crataegi fructus] (Shanzha), 720g<br><br>Made into 1,000 tablets	Y*	Y-HPLC (Yang, 2012), HPLC (Zhang et al., 2012)
Huazhi Rougan granule	Shandong New Era Pharmaceutical Co. Ltd.	Z20090077	*Artemisia capillaris* Thunb. [Asteraceae; Artemisiae scopariae herba] (Yinchen); *Senna tora* (L.) Roxb. [Fabaceae; Cassiae semen] (Juemingzi); *Rheum palmatum* L. [Polygonaceae; Rhei radix et rhizoma] (Dahuang); *Alisma plantago-aquatica subsp. orientale* (Sam.) Sam. [Alismataceae; Alismatis rhizoma] (Zexie); *Crotalaria albida* B.Heyne ex Roth [Fabaceae; Polyporus] (Zhuling); *Crataegus pinnatifida* Bunge [Rosaceae; Crataegi fructus] (Shanzha); *Atractylodes lancea* (Thunb.) DC. [Asteraceae; Atractylodis rhizoma] (Cangzhu); *Atractylodes macrocephala* Koidz. [Asteraceae; Atractylodis macrocephalae rhizoma] (Baizhu); *Citrus × aurantium* L. [Rutaceae; Citri reticulatae pericarpium] (Chenpi); *Trichosanthes kirilowii* Maxim. [Cucurbitaceae; Trichosanthis fructus] (Gualou); *Ligustrum lucidum* W.T.Aiton [Oleaceae; Ligustri lucidi fructus] (Nvzhenzi); *Eclipta prostrata* (L.) L. [Asteraceae; Ecliptae herba] (Mohanlian); *Lycium barbarum* L. [Solanaceae; Lycii fructus] (Gouqizi); *Cirsium japonicum* DC. [Asteraceae; Cirsii herba] (Xiaoji); *Bupleurum chinense* DC. [Apiaceae; Bupleuri radix] (Chaihu); *Glycyrrhiza glabra* L. [Fabaceae; Glycyrrhizae radix et rhizoma] (Gancao)<br><br>Unknown dosage	Y*	N
Qiaozhi capsule	Inner Mongolia Furui	Z20050665	Chinemys reevesii (Gray) [Tortoidae; Testudinis carapax et plastrum] (Guijia); *Reynoutria multiflora* (Thunb.) Moldenke [Polygonaceae; Polygoni multiflori radix praeparata] (Zhiheshouwu); *Artemisia capillaris* Thunb. [Asteraceae; Artemisiae	Y*	N

	Medical Technology Co. Ltd.		scopariae herba] (Yinchen); *Salvia miltiorrhiza* Bunge [Lamiaceae; Salviae miltiorrhizae radix et rhizoma] (Danshen); *Achyranthes bidentata* Blume [Amaranthaceae; Achyranthis bidentatae radix] (Niuxi);<br><br>Unknown dosage		
Sanqi Zhigan pill	Yunnan Yuyao Biopharmaceutical Co. Ltd.	Z20025353	*Panax notoginseng* (Burkill) F.H.Chen [Araliaceae; Notoginseng radix et rhizoma] (Sanqi); *Curcuma longa* L. [Zingiberaceae; Curcumae rhizoma] (Ezhu); *Crataegus pinnatifida* Bunge [Rosaceae; Crataegi fructus] (Shanzha); *Alisma plantago-aquatica subsp. orientale* (Sam.) Sam. [Alismataceae; Alismatis rhizoma] (Zexie); *Chrysanthemum × morifolium* (Ramat.) Hemsl. [Asteraceae; Chrysanthemi flos] (Juhua); *Nelumbo nucifera* Gaertn. [Nymphaeaceae; Nelumbinis folium] (Heye); *Paeonia lactiflora* Pall. [Paeoniaceae; Paeoniae radix alba] (Baishao); *Atractylodes macrocephala* Koidz. [Asteraceae; Atractylodis macrocephalae rhizoma] (Baizhu); *Cuscuta chinensis* Lam. [Convolvulaceae; Cuscutae semen] (Tusizi); *Paeonia lactiflora* Pall. [Paeoniaceae; Paeoniae radix rubra] (Chishao); *Citrus × aurantium* L. [Rutaceae; Citri reticulatae pericarpium viride] (Qingpi)<br><br>Unknown dosage	Y*	Y-HPLC (Li et al., 2015)
Liuwei Wuling tablet	Shandong Shibo Jindu Pharmaceutical Co. Ltd.	Z20060238	*Schisandra chinensis* (Turcz.) Baill. [Schisandraceae; Schisandrae chinensis fructus] (Wuweizi); *Ligustrum lucidum* W.T.Aiton [Oleaceae; Ligustri lucidi fructus] (Nvzhenzi); *Forsythia suspensa* (Thunb.) Vahl [Oleaceae; Forsythiae fructus] (Lianqiao); *Curcuma longa* L. [Zingiberaceae; Curcumae rhizoma] (Ezhu); *Cichorium intybus* L. [Asteraceae; Cichorii herba cichorii radix] (Juju); *Ganoderma lucidum* (Leyss.ex Fr.) Karst. [Poromycelidae; Ganoderma] (Lingzhi)<br><br>Unknown dosage	Y*	Y-HPLC-DAD (Ling, 2017), HPLC (Cui et al., 2016)
Hedan tablet	Jiangxi Wohua Jishun	Z20023129	*Nelumbo nucifera* Gaertn. [Nelumbonaceae; Nelumbinis folium] (Heye), 7500g; *Salvia miltiorrhiza* Bunge [Lamiaceae; Salviae miltiorrhizae radix et rhizoma] (Danshen), 1250g; *Crataegus pinnatifida* Bunge [Rosaceae; Crataegi fructus] (Shanzha), 3750g;	Y*	Y-DMSPE-HPLC (Liu et

	Pharmaceutical Co. Ltd.		*Senna alexandrina* Mill. [Fabaceae; Sennae folium] (Fanxieye), 375g; *Cullen corylifolium* (L.) Medik. [Fabaceae; Psoraleae fructus] (Buguzhi) 1250g<br><br>Made into 2,500 tablets		al., 2021)
Gandan Shukang capsule	Henan Runhong Pharmaceutical Co. Ltd.	B20020053	*Paeonia lactiflora* Pall. [Paeoniaceae; Paeoniae radix alba] (Baishao); *Artemisia capillaris* Thunb. [Asteraceae; Artemisiae scopariae herba] (Yinchen); *Bupleurum chinense* DC. [Apiaceae; Bupleuri radix] (Chaihu); *Curcuma longa* L. [Zingiberaceae; Curcumae radix] (Yujin); *Salvia miltiorrhiza* Bunge [Lamiaceae; Salviae miltiorrhizae radix et rhizoma] (Danshen); Trionyx sinensis Wiegmann [Trionychiaceae; Trionycis carapax] (Biejia); *Ziziphus jujuba* Mill. [Rhamnaceae; Jujubae fructus] (Dazao)<br><br>Unknown dosage	Y*	N
Xuezhikang capsule	Beijing Beida Weixin Biotechnology Co. Ltd.	Z10950029	Monascus purpureus Went. [Aspergillaceae; Monascus] (Hongqu)<br><br>Unknown dosage	Y*	Y-HPLC-DAD (Li and Sun, 2019)
Yiganling tablet	Shanghai Chaohui Pharmaceutical Co. Ltd.	Z31020107	*Silybum marianum* (L.) Gaertn. [Asteraceae; Silybi fructus] (Shuifeiji)<br><br>Unknown dosage	Y*	Y-HPLC (Liang et al., 2008)
Hugan capsule	Nanjing Tongrentang Pharmaceutical Co. Ltd.	Z20027711	*Bupleurum chinense* DC. [Apiaceae; Bupleuri radix] (Chaihu), 313g; *Artemisia capillaris* Thunb. [Asteraceae; Artemisiae scopariae herba] (Yinchen), 313g; *Isatis tinctoria* L. [Brassicaceae; Isatidis radix] (Banlangen), 313g; *Schisandra chinensis* (Turcz.) Baill. [Schisandraceae; Schisandrae chinensis fructus] (Wuweizi), 168g; Sus scrofa domestica Brisson. [Suidae; Suis fellis pulvis] (Zhudanfen), 20g; *Vigna umbellata* (Thunb.) Ohwi & H.Ohashi [Fabaceae; Vignae semen] (Chixiaodou), 128g	Y*	N

			Made into 1,000 capsules		
Zhibitai capsule	Chengdu Dior Jiuhong Pharmaceutical Factory	Z51022196	*Crataegus pinnatifida* Bunge [Rosaceae; Crataegi fructus] (Shanzha); *Alisma plantago-aquatica subsp. orientale* (Sam.) Sam. [Alismataceae; Alismatis rhizoma] (Zexie); *Atractylodes macrocephala* Koidz. [Asteraceae; Atractylodis macrocephalae rhizoma] (Baizhu); Monascus purpureus Went. [Aspergillaceae; Monascus] (Hongqu)<br><br>Unknown dosage	Y*	Y-HPLC (Wen et al., 2011), HPLC

Notes: CPMs, Chinese patent medicines; SFDA, State Food and Drug Administration; *, Prepared according to People's Republic of China Pharmacopoeia.

References

Cui, H., Xu, G., Wu, M., Jiang, W., Han, J., Wang, J., et al. (2016). Simultaneous Determination of Eight Active Components in Liuwei Wuling Tablet Using HPLC. Chin Herb Med. 8 (4), 331-336. doi: 10.1016/s1674-6384(16)60059-x

Li L. L., Zhou L. (2015). Determination of the Contents of Ginsenoside Rg1 in Sanqizhigan Pills by HPLC. Strait Phar J. 27(12), 39-41.

Li Y., Sun X. (2019) Simultaneous Content Determination of 8 Components in Xuezhikang Capsules by HPLC-DAD. Chin Ph. 30(15), 2066-2070.

Liang M., Song F. X., Lv C. X. (2008). Contentan alysis of silybinin Yiganling tablets by RP-HPLC. J Guangdong Colg Ph. 24(05), 472-474. doi:10.16809/j.cnki.1006-8783.2008.05.01

Ling, C. (2017). Simultaneous determination of seven bioactive constituents in Liuwei Wuling tablet by HPLC coupled with DAD. Jcps. 26 (4), 304-311. doi: 10.5246/jcps.2017.04.032

Liu Y. J., Yu H. M., Zhang H., Chen Q., Chen D. W. (2021). Dispersive Micro Solid-Phase Extraction Coupled with HPLC-UV for Detection of Nuciferine, N-nornuciferine, O-nornuciferine in He-Dan-Pian. J Fujian Med Univ. 55(03), 224-229.

Long Y. T., Chen H., Liu J. (2017). Simultaneous determination of seven constituents in Dangfei Liganning Capsules by HPLC. Chin Tradit Pat Med. 39(06), 1187-1190.

Ma, X. W., Wei, H., Liu S. X., Liu Y., Zhang T. J., Chen C. Q. (2012). Determination of gentiopicroside, paeoniflorin, and salvianolic acid B in Qianggan Capsule by HPLC. Chin Tradit Herb Drugs. 43(06), 1125-1128.

Pan R. L., Han H. Q. (2018). Determination of Dangfeiliganning Capsules by HPLC. World Latest Med Info. 18(34), 221-222. doi:10.19613/j.cnki.1671-3141.2018.34.118

Wei H., Liu S. X., Liu Y., Zhang T. J., Chen C. Q. (2013). HPLC fingerprint of Qianggan Capsule. Chin Tradit Herb Drugs. 44(07), 845-850.

Wen Z. M., Zhang F., Huang Y., Luo J. X., Wei Z. X., et al. (2011). Determination of Lovastatin in Zhibitai Capsules by HPLC. Chin Phar. 44(07), 845-850.
Yang K. (2012). Determination of the content of herba lysimachiae in danning tablet by high performance liquid chromatographic method. Clin J Chin Med. 4(23), 34-35.
Zhang Q. R., Wang L. F., Liu W. Y. (2012). HPLC Simultaneous Determination of Five Kinds of Anthraquinones in Danning Tablets. Chin J PV. 9(01), 3-6.

4 Forest plots

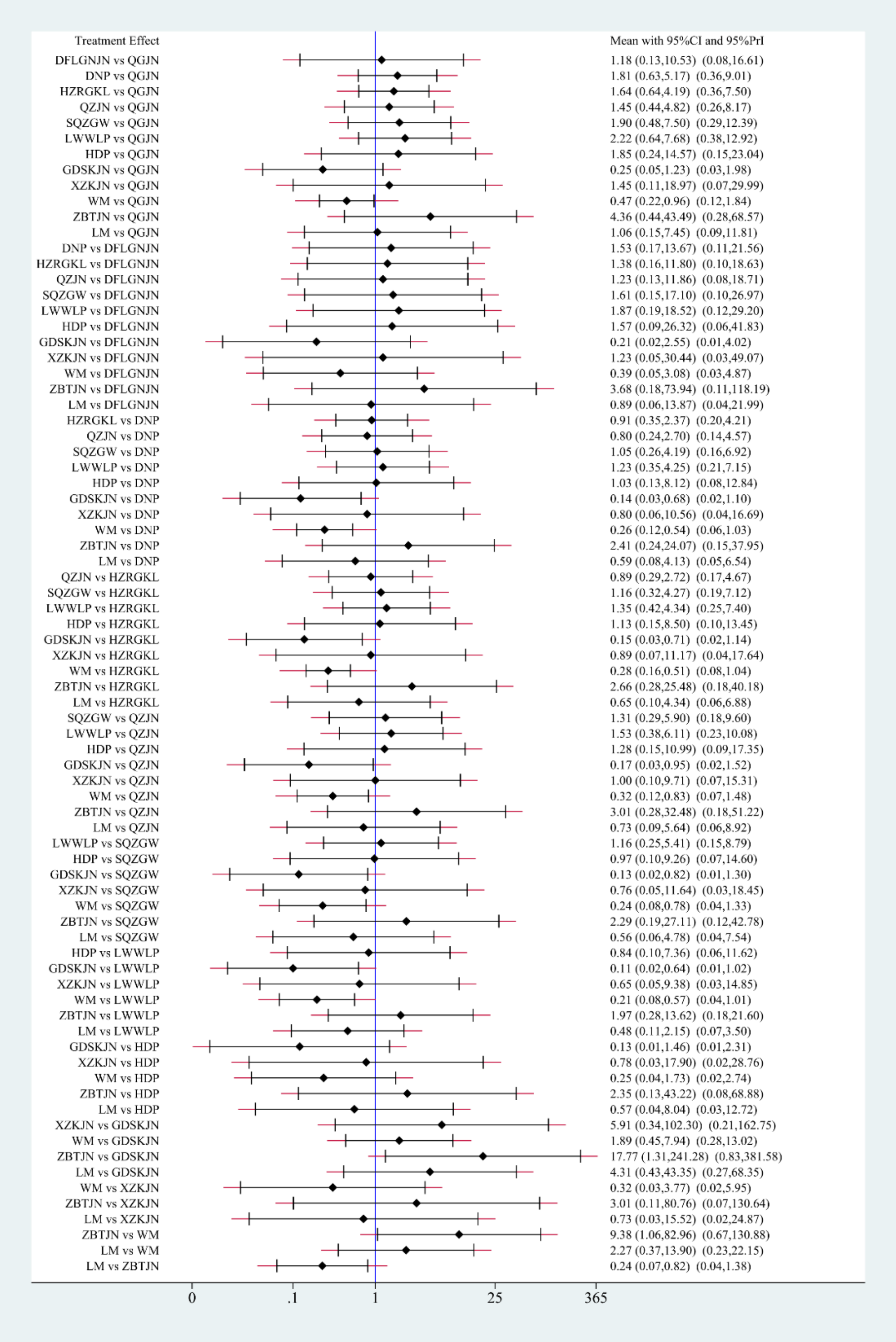

Figure S1. Forest plot in clinical efficiency rate. QGJN, Qianggan capsule; DFLGNJN, Dangfei Liganning capsule; DNP, Danning tablet; HZRGKL, Huazhi Rougan granule; QZJN, Qiaozhi capsule; SQZGW, Sanqi Zhigan pill; LWWLP, Liuwei Wuling tablet; HDP, Hedan tablet; GDSKJN, Gandan Shukang capsule; XZKJN, Xuezhikang capsule; WM, Western medicine; ZBTJN, Zhibitai capsule; LM, lifestyle modification.

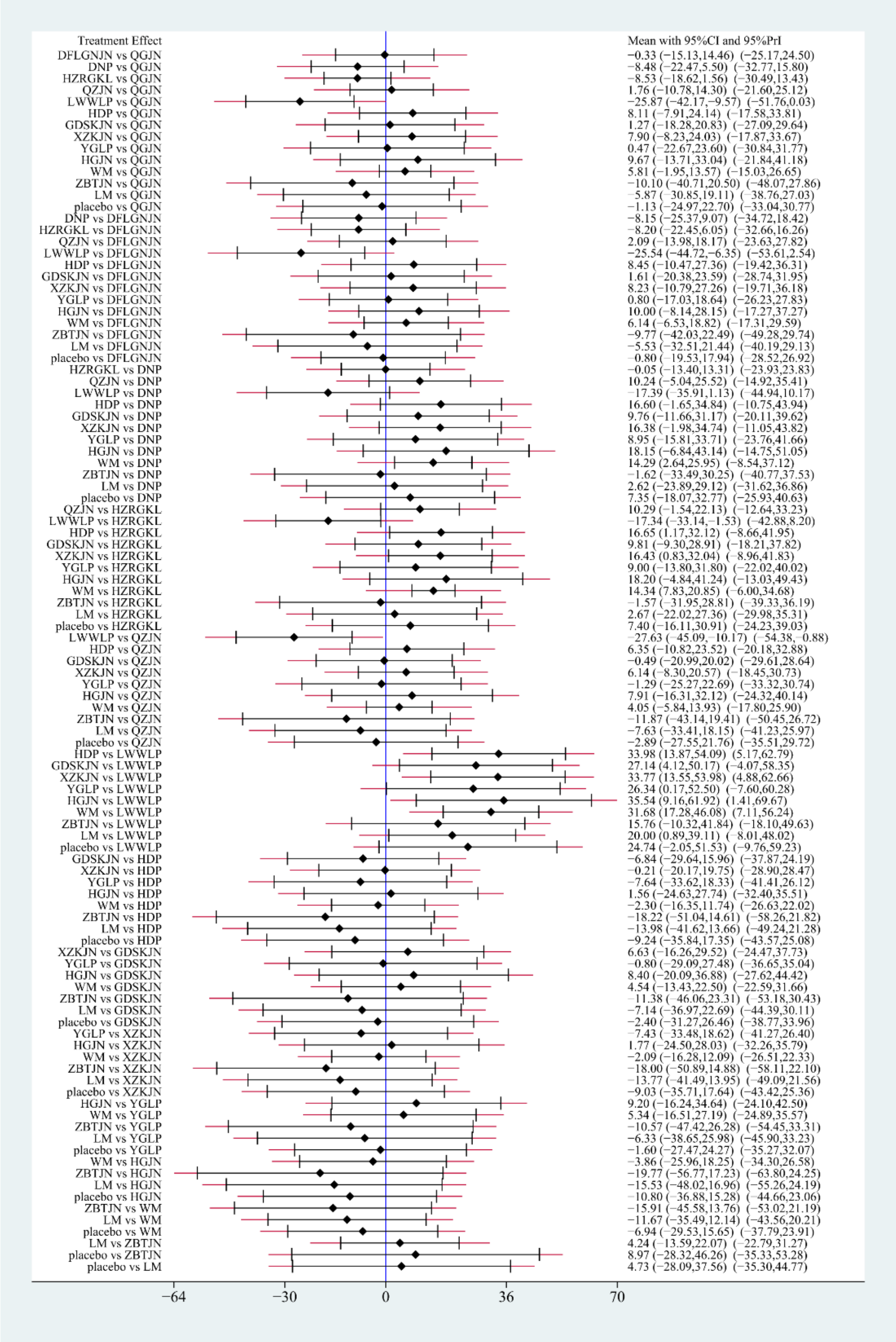

Figure S2. Forest plot in ALT.QGJN, Qianggan capsule; DFLGNJN, Dangfei Liganning capsule; DNP, Danning tablet; HZRGKL, Huazhi Rougan granule; QZJN, Qiaozhi capsule; LWWLP, Liuwei Wuling tablet; HDP, Hedan tablet; GDSKJN, Gandan Shukang capsule; XZKJN, Xuezhikang capsule; YGLP, Yiganling tablet; HGJN, Hugan capsule; WM, Western medicine; ZBTJN, Zhibitai capsule; LM, lifestyle modification.

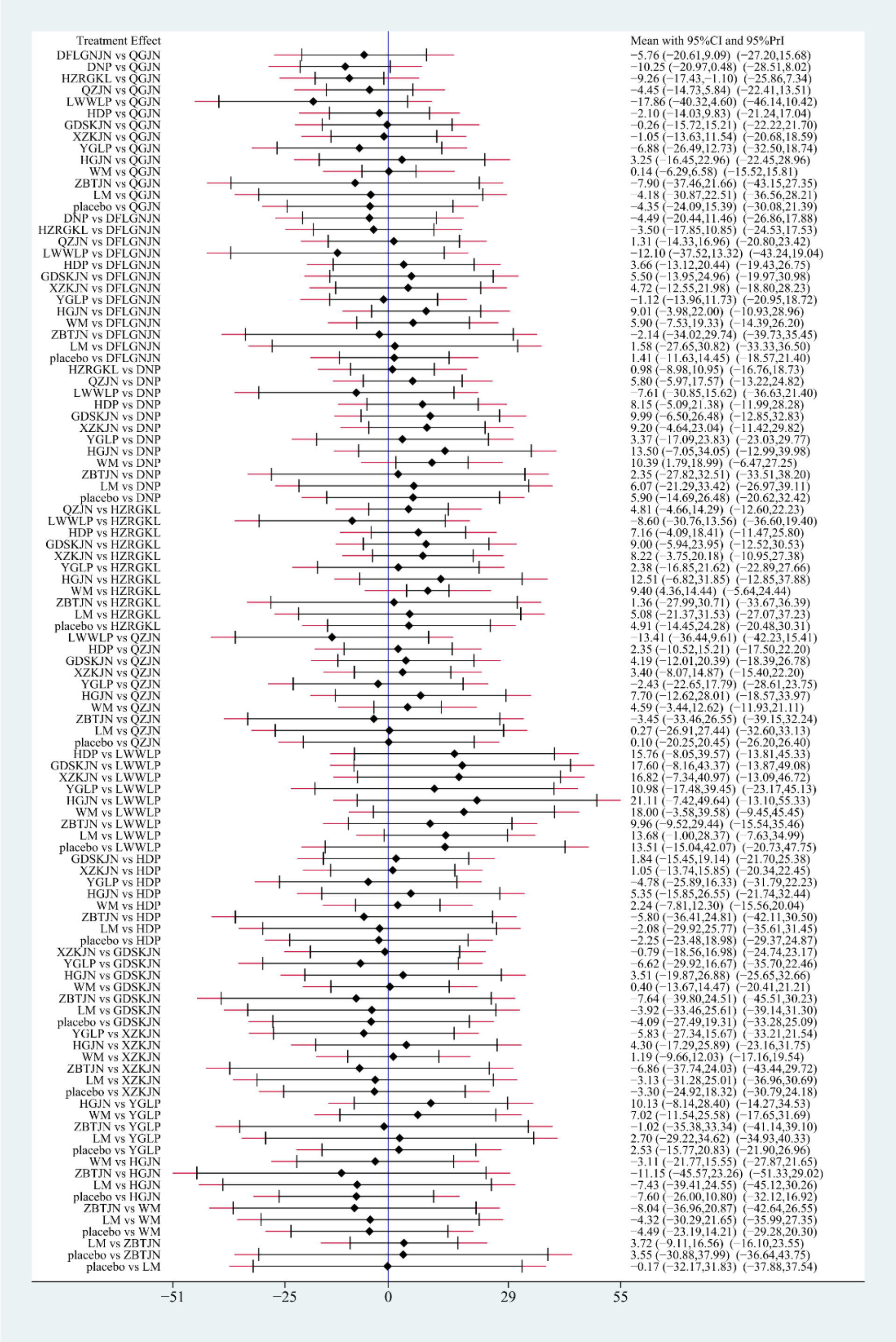

Figure S3. Forest plot in AST. QGJN, Qianggan capsule; DFLGNJN, Dangfei Liganning capsule; DNP, Danning tablet; HZRGKL, Huazhi Rougan granule; QZJN, Qiaozhi capsule; LWWLP, Liuwei Wuling tablet; HDP, Hedan tablet; GDSKJN, Gandan Shukang capsule; XZKJN, Xuezhikang capsule; YGLP, Yiganling tablet; HGJN, Hugan capsule; WM, Western medicine; ZBTJN, Zhibitai capsule; LM, lifestyle modification.

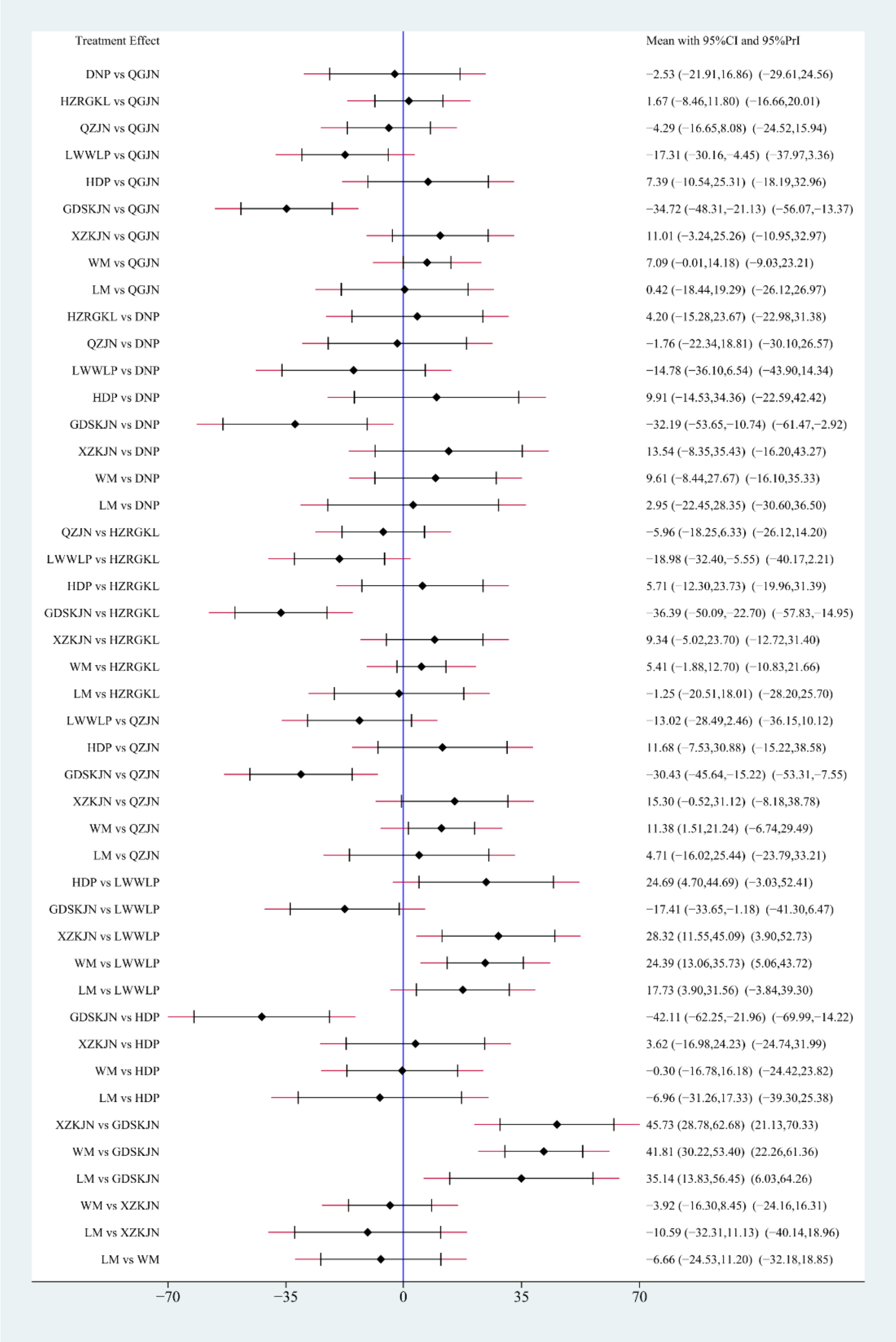

Figure S4. Forest plot in GGT. QGJN, Qianggan capsule; DNP, Danning tablet; HZRGKL, Huazhi Rougan granule; QZJN, Qiaozhi capsule; LWWLP, Liuwei Wuling tablet; HDP, Hedan tablet; GDSKJN, Gandan Shukang capsule; XZKJN, Xuezhikang capsule; WM, Western medicine; ZBTJN, Zhibitai capsule; LM, lifestyle modification.

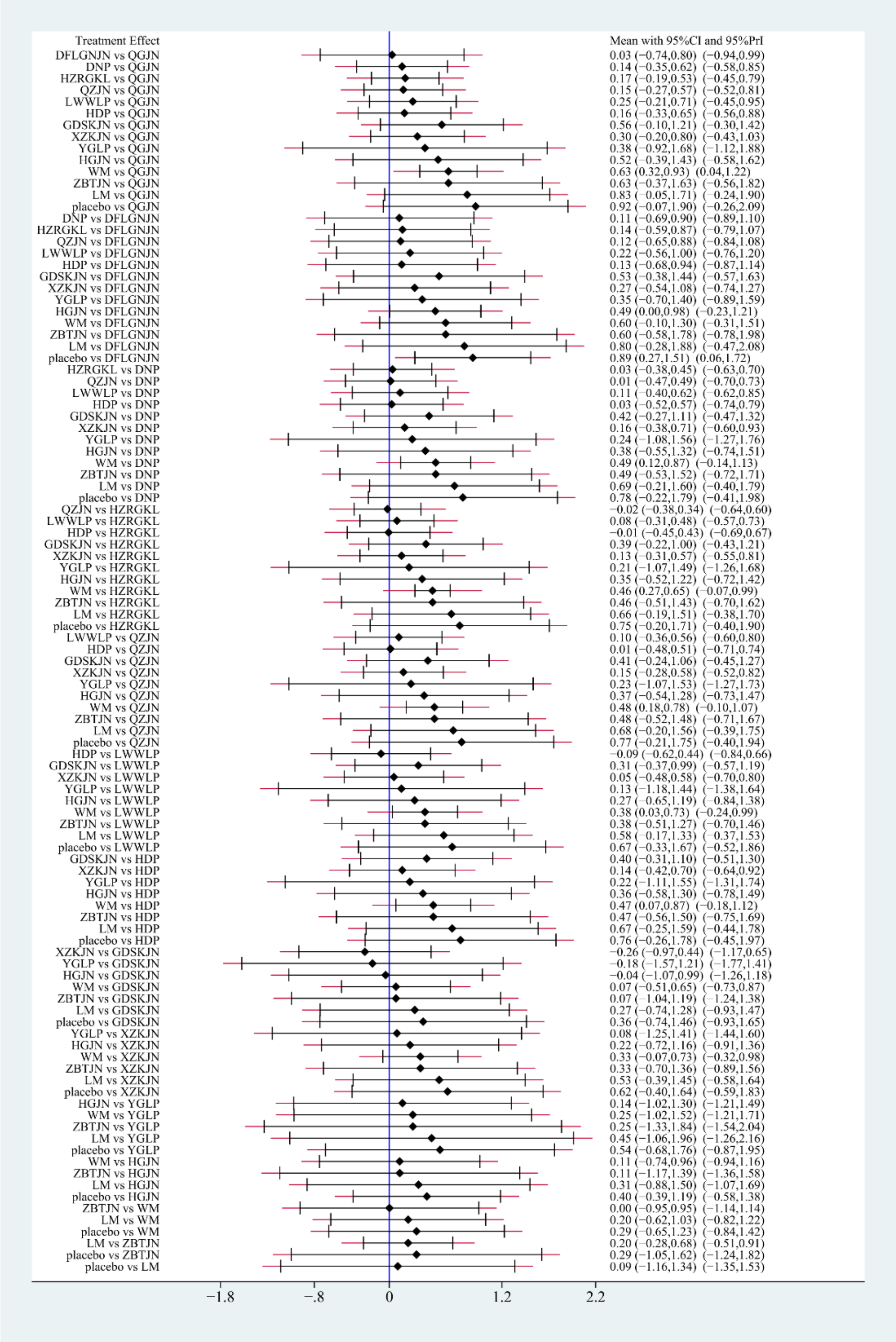

Figure S5. Forest plot in TG. QGJN, Qianggan capsule; DFLGNJN, Dangfei Liganning capsule; DNP, Danning tablet; HZRGKL, Huazhi Rougan granule; QZJN, Qiaozhi capsule; LWWLP, Liuwei Wuling tablet; HDP, Hedan tablet; GDSKJN, Gandan Shukang capsule; XZKJN, Xuezhikang capsule; YGLP, Yiganling tablet; HGJN, Hugan capsule; WM, Western medicine; ZBTJN, Zhibitai capsule; LM, lifestyle modification.

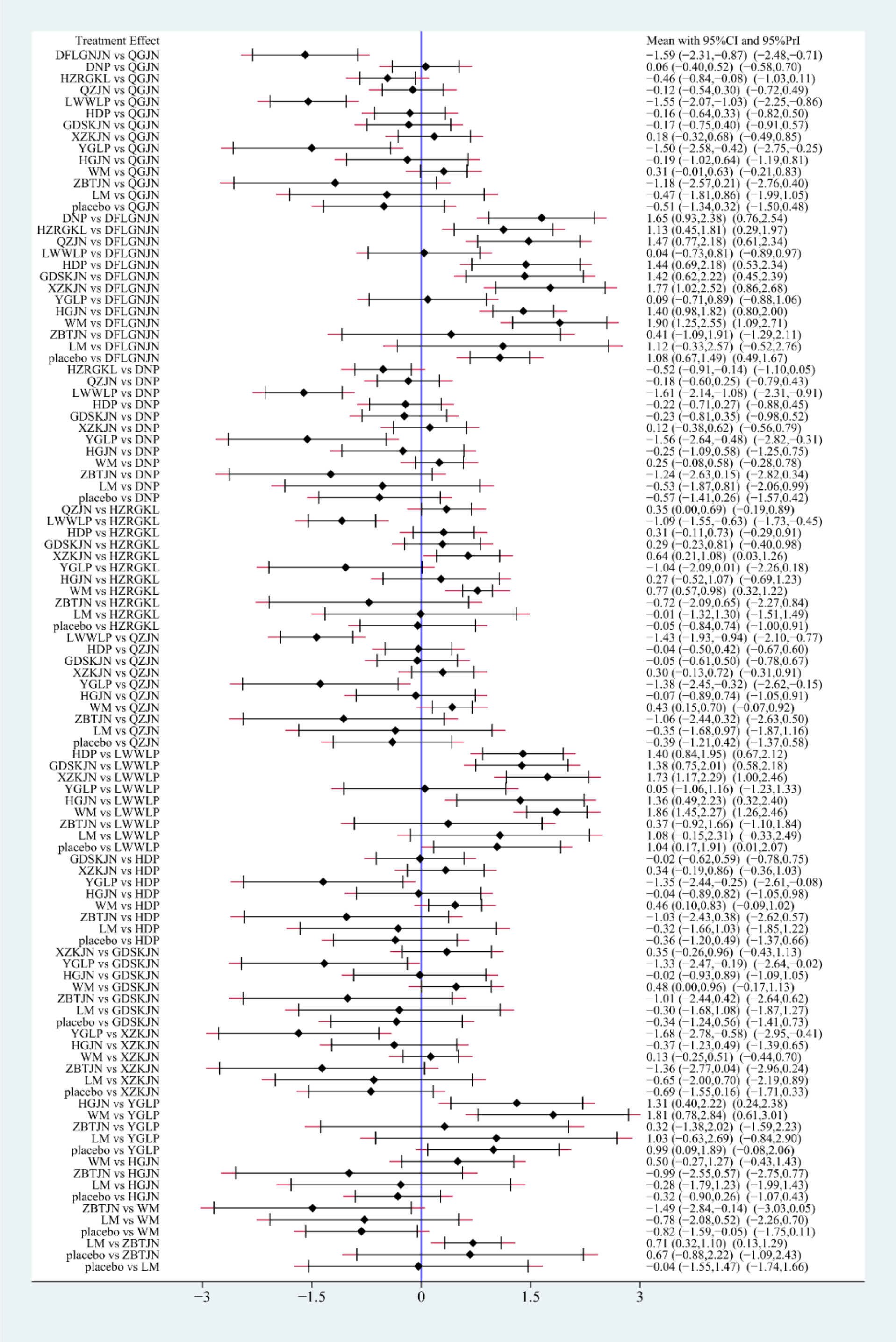

Figure S6. Forest plot in TC. QGJN, Qianggan capsule; DFLGNJN, Dangfei Liganning capsule; DNP, Danning tablet; HZRGKL, Huazhi Rougan granule; QZJN, Qiaozhi capsule; LWWLP, Liuwei Wuling tablet; HDP, Hedan tablet; GDSKJN, Gandan Shukang capsule; XZKJN, Xuezhikang capsule; YGLP, Yiganling tablet; HGJN, Hugan capsule; WM, Western medicine; ZBTJN, Zhibitai capsule; LM, lifestyle modification.

5 Adverse reactions

Table10 Adverse reactions

**Study ID**	**Intervention of Experimental group**	**Intervention of Control group**	**Adverse reactions in Experimental group**	**Adverse reactions in Control group**
Chen ZX 2006	QGJN	WM	NR	NR
Li L 2010	QGJN	WM	Nausea occurred in 2 cases, burning sensation in 3 cases, and diarrhea in 1 case.	1 case of nausea and 1 case of diarrhea.
Ou Q 2016	QGJN	WM	N	N
He ZJ 2016	QGJN	WM	N	N
Wang YP 2015	QGJN	WM	NR	NR
Liu ZY 2014	QGJN	WM	NR	NR
Wu J 2018	DFLGNJN	WM	NR	NR
Wu JY 2008	DFLGNJN	WM	NR	NR
Ji G 2008	DNP	WM	1 case had skin rash, 3 cases had nausea, and most patients had increased stool frequency, but they could tolerate them or relieved after drug reduction.	N
Lv QG 2009	DNP	WM	NR	NR
Wang ZL 2014	DNP	WM	The stool frequency of most patients increased,	N

			and most of them relieved spontaneously after drug reduction, but 4 cases needed to be controlled with antidiarrheal drugs; Nausea occurred in 5 cases; 3 cases had mild abdominal pain, but they could tolerate it or relieved after drug reduction.	
Ma JY 2014	DNP	WM	NR	NR
Yang SS 2015	HZRGKL	WM	N	Nausea and diarrhea occurred in 5 cases, and blood pressure increased in 5 cases.
Wei B 2015	HZRGKL	WM	NR	NR
Li HX 2017	HZRGKL	WM	N	N
Wang XL 2018	HZRGKL	WM	N	N
Zhang CM 2019	HZRGKL	WM	NR	NR
Lin YD 2013	HZRGKL	WM	NR	NR
Yu Y 2014	HZRGKL	WM	1 case had epigastric discomfort and 1 case had mild diarrhea. They were untreated but relieved.	1 case had slight abdominal distension and 1 case had slight dizziness. They were untreated but relieved.
Xu JL 2018	HZRGKL	WM	NR	NR
Nan C	HZRGKL	WM	NR	NR

2020				
Meng SX 2009	QZJN	WM	1 case of abdominal distension and 1 case of anorexia; They were not treated, but they relieved themselves.	N
Xu L 2010	QZJN	WM	One case developed abdominal distension, and recovered after 2 days of drug withdrawal, without abdominal distension.	N
Ning HM 2017	QZJN	WM	1 case had mild stomach discomfort, 2 cases had diarrhea. Symptoms disappeared after symptomatic treatment.	Mild gastric discomfort occurred in 2 cases and disappeared after symptomatic treatment.
Zhang JM 2011	SQZGW	WM	NR	NR
Luo D 2014	SQZGW	WM	NR	NR
Liu M 2011	LWWLP	WM	NR	NR
Zhang XL 2014	LWWLP	WM	N	N
Li FB 2009	HDP	WM	N	N
Deng YQ 2011	HDP	WM	1 case developed nausea, but the symptom disappeared after taking medicine after meals; Stool frequency increased in 1 case and disappeared after drug dosage decreased.	N
Yuan L	GDSKJN	WM	N	N

2008				
Fan XF 2010	XZKJN	WM	One case suffered from diarrhea, relieved after drug withdrawal, but withdrew from the study, which was a lost case.	N
Huang X 2007	DFLGNJN	YGLP	NR	NR
Qi M 2012	DFLGNJN	HGJN	NR	NR
Yang YW 2014	QZJN	XZKJN	N	N
Li CM 2012	DFLGNJN	placebo	N	N
Peng J 2019	ZBTJN	LM	N	N
Jin YR 2011	LWWLP	LM	N	N
Zhong ZM 2017	ZBTJN	LM	N	N

Note: QGJN, Qianggan capsule; DFLGNJN, Dangfei Liganning capsule; DNP, Danning tablet; HZRGKL, Huazhi Rougan granule; QZJN, Qiaozhi capsule; SQZGW, Sanqi Zhigan pill. LWWLP, Liuwei Wuling tablet; HDP, Hedan tablet; GDSKJN, Gandan Shukang capsule; XZKJN, Xuezhikang capsule; YGLP, Yiganling tablet; HGJN, Hugan capsule; WM, Western medicine; ZBTJN, Zhibitai capsule; LM, lifestyle modification. NR: not reported; N: no.

6 Inconsistency plots

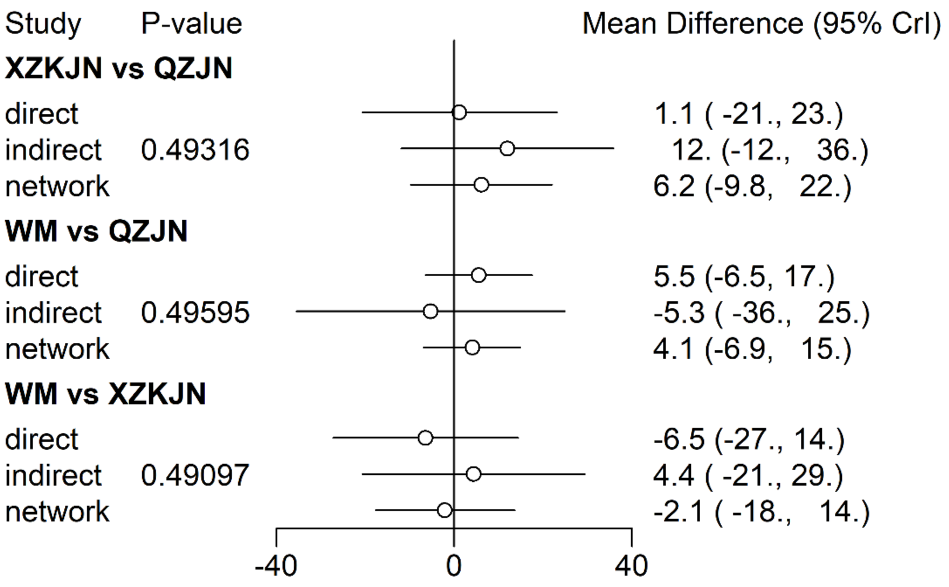

Figure S7. Inconsistency plot in ALT. XZKJN, Xuezhikang capsule; QZJN, Qiaozhi capsule; WM, Western medicine.

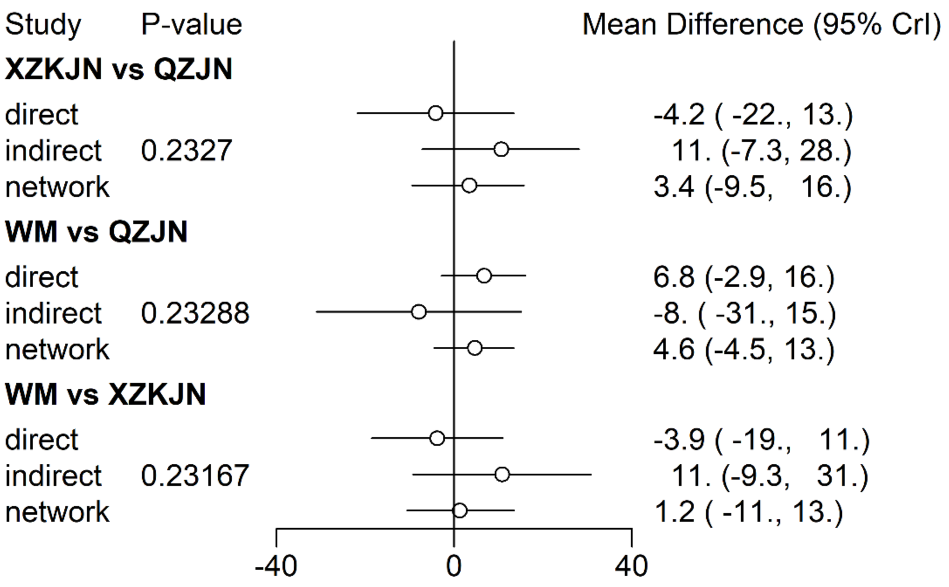

Figure S8. Inconsistency plot in AST.XZKJN, Xuezhikang capsule; QZJN, Qiaozhi capsule; WM, Western medicine.

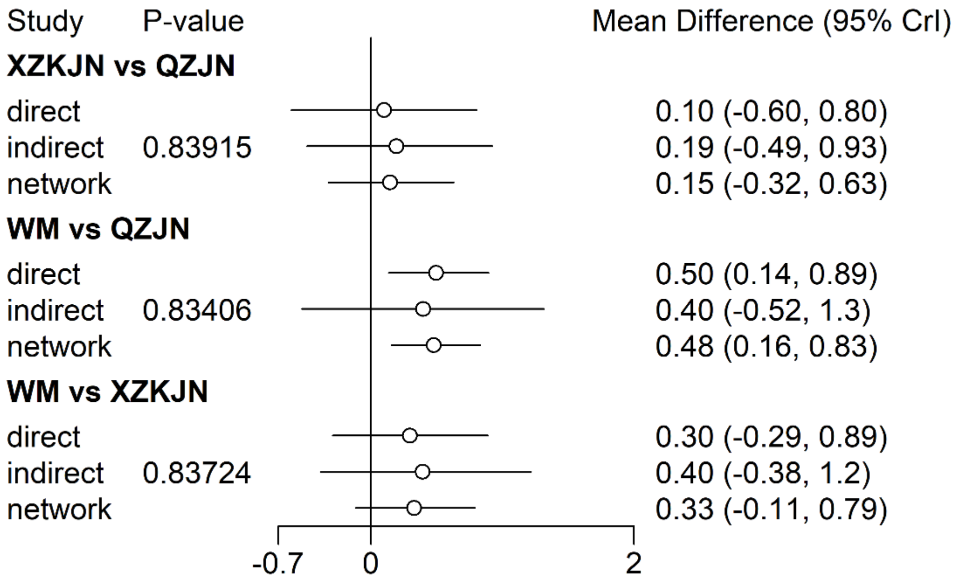

Figure S9. Inconsistency plot in TG. XZKJN, Xuezhikang capsule; QZJN, Qiaozhi capsule; WM, Western medicine.

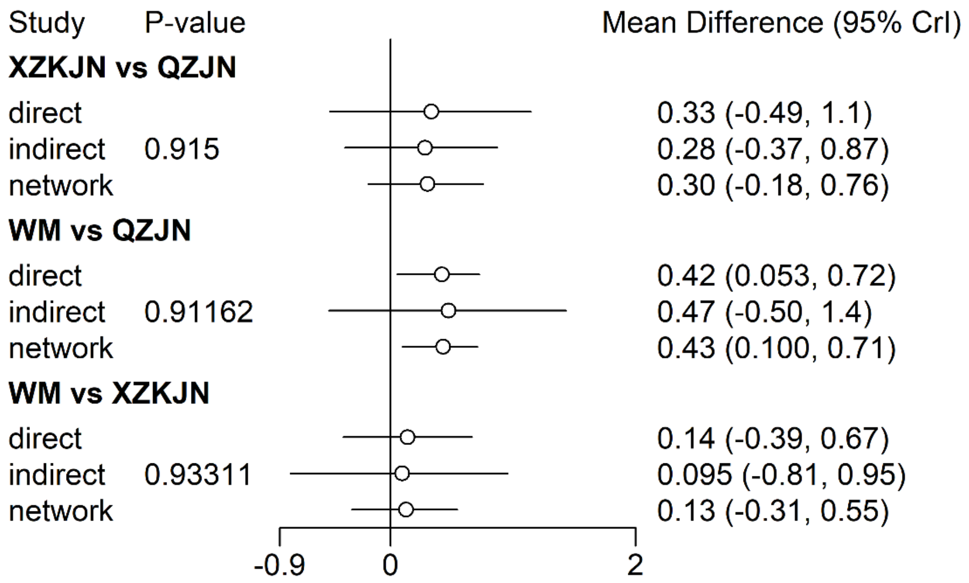

Figure S10. Inconsistency plot in TC. XZKJN, Xuezhikang capsule; QZJN, Qiaozhi capsule; WM, Western medicine.

7 Heterogeneity analysis

Table 11 heterogeneity analysis

	clinical efficiency rate (Global I^2=54.2%)	ALT(Global I^2=94.2%)	AST (Global I^2=91.1%)	GGT (Global I^2=73.6%)	TG(Global I^2=80.3%)	TC(Global I^2=44.2%)
Pairwise comparison	Per-comparisonI^2	Per-comparisonI^2	Per-comparisonI^2	Per-comparisonI^2	Per-comparisonI^2	Per-comparisonI^2
DFLGNJN vs HGJN	-	-	-	-	-	-
DFLGNJN vs WM	-	95.7%	-	-	-	-
DFLGNJN vs YGLP	-	-	-	-	-	-
DNP vs WM	89.7%	85.7%	80.3%	-	72.0%	0.0%
GDSKJN vs WM	-	-	-	-	-	-
HDP vs WM	-	82.8%	0.0%	-	77.1%	0.0%
HZRGKL vs WM	0.0%	96.8%	95.4%	44.4%	83.1%	33.5%
LWWLP vs WM	0.0%	0.0%	-	89.2%	8.7%	-
QGJN vs WM	0.0%	77.7%	0.0%	61.4%	80.7%	0.0%
QZJN vs WM	0.0%	96.0%	90.1%	86.1%	90.2%	86.9%

QZJN vs XZKJN	-	-	-	-	-	-
WM vs XZKJN	-	-	-	-	-	-
SQZGW vs WM	0.0%	-	-	-	-	-

Note: QGJN, Qianggan capsule; DFLGNJN, Dangfei Liganning capsule; DNP, Danning tablet; HZRGKL, Huazhi Rougan granule; QZJN, Qiaozhi capsule; SQZGW, Sanqi Zhigan pill. LWWLP, Liuwei Wuling tablet; HDP, Hedan tablet; GDSKJN, Gandan Shukang capsule; XZKJN, Xuezhikang capsule; YGLP, Yiganling tablet; HGJN, Hugan capsule; WM, Western medicine; ZBTJN, Zhibitai capsule; LM, lifestyle modification.

8 Sensitivity analysis

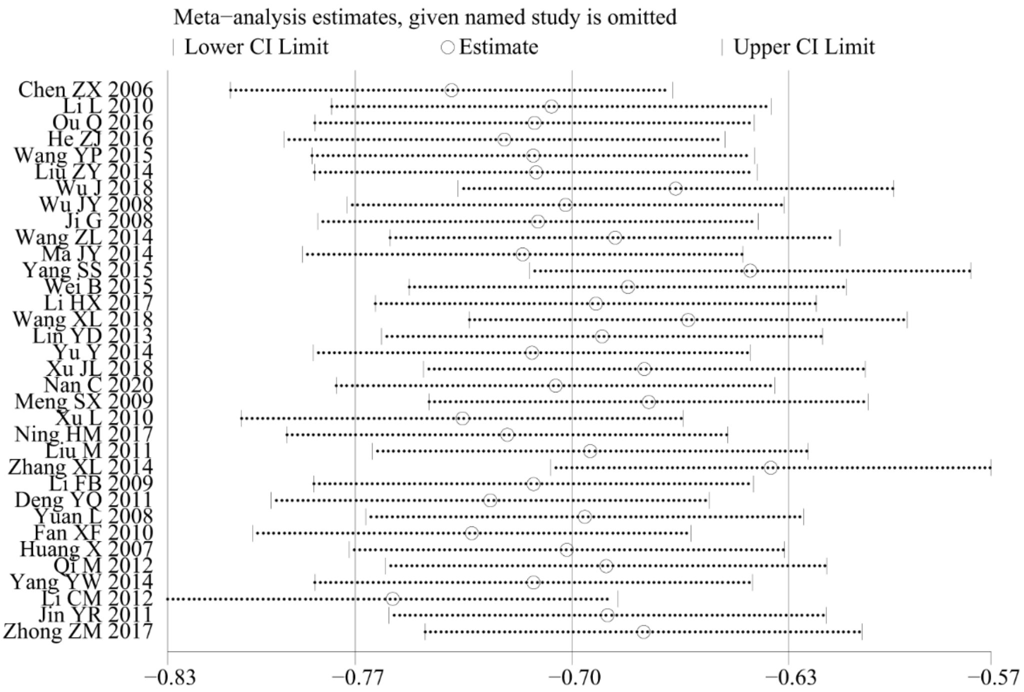

Figure S11 Sensitivity analysis of ALT.

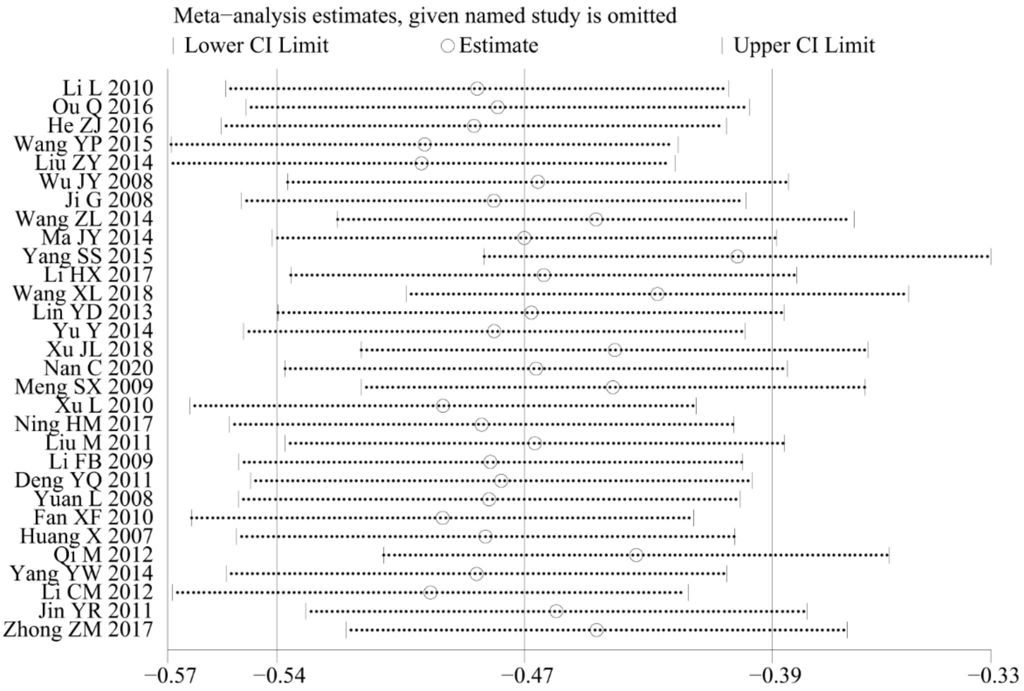

Figure S12 Sensitivity analysis of AST.

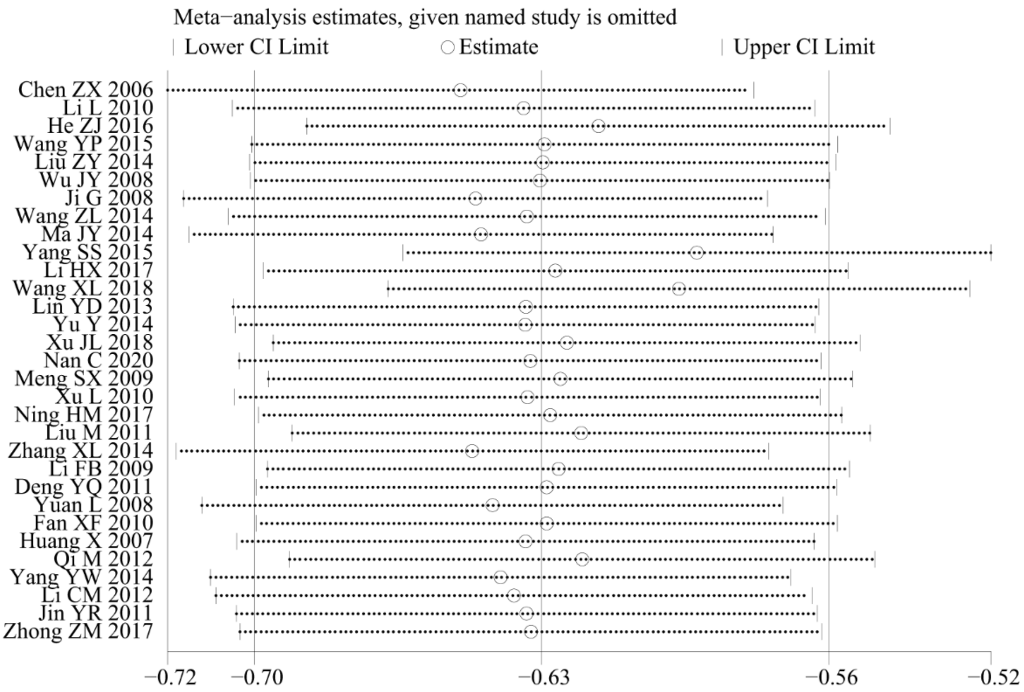

Figure S13 Sensitivity analysis of TG.
